# A SUMO interacting motif in the replication initiator protein of tomato yellow leaf curl virus is required for viral replication

**DOI:** 10.1128/jvi.01286-25

**Published:** 2025-11-10

**Authors:** Nicolas Frédéric Gaertner, Francesca Maio, Manuel Arroyo-Mateos, Ana P. Luna, Blanca Sabarit, Mark Kwaaitaal, Sandra Eltschkner, Marcel Prins, Eduardo R. Bejarano, Harrold A. van den Burg

**Affiliations:** 1 Molecular Plant Pathology, Swammerdam Institute for Life Sciences, research theme Green Life Sciences, Faculty of Science, University of Amsterdam 1234, Amsterdam, the Netherlands; 2 Dept. Biología Celular, Genética y Fisiología, Instituto de Hortofruticultura Subtropical y Mediterránea “La Mayora”, Universidad de Málaga-Consejo Superior de Investigaciones Científicas (IHSM-UMA-CSIC), Universidad de Málaga 16752, Málaga, Spain; 3 Keygene N.V., Wageningen, the Netherlands; 4 Rijk Zwaan Breeding B.V., De Lier, the Netherlands; Tsinghua University, Beijing, China

**Keywords:** CRESS-DNA virus, geminivirus, Rep, C1, SUMO, SCE1, TYLCV, TGMV, DNA replication, ATPase activity, begomovirus, systemic movement, virus replication

## Abstract

Circular Rep-encoding single-stranded (CRESS)-DNA viruses comprise a diverse group of viruses that use rolling-circle replication to copy their genomes. They infect organisms in almost all branches of the eukaryotic tree of life. A hallmark of all CRESS-DNA viruses is the presence of a single conserved protein, the replication initiator protein (Rep), that orchestrates viral replication by exploiting the host DNA replication machinery. In the case of the plant-infecting *Geminiviridae*, this multifunctional protein both recruits the host DNA replication machinery and manipulates post-translational modification including small ubiquitin-like modifier (SUMO) conjugation. In fact, Rep from two different geminiviruses, tomato yellow leaf curl virus (TYLCV; *Begomovirus coheni*) and tomato golden mosaic virus (TGMV; *B. solanum aureimusivi*), was earlier shown to interact with the SUMO conjugating enzyme SCE1. Here, we demonstrate that TYLCV C1/Rep protein also interacts with Arabidopsis SUMO1 and identify a SUMO interacting motif (SIM) located in the C-terminal SF3 helicase/ATPase domain. Remarkably, a functional SIM proved to be important for the interaction of Rep with both SUMO1 and SCE1. The same motif in the Rep ORF was essential for TYLCV viral replication, disease symptom formation, and systemic movement from an infectious clone, and Rep ATPase activity. Together, our findings thus connect the interaction between Rep and the SUMO machinery with TYLCV viral replication.

**IMPORTANCE:**

The identification of a non-canonical SUMO-interacting motif (SIM) within the Rep protein of tomato yellow leaf curl virus (TYLCV) reveals a connection between viral replication and a protein modification, i.e., SUMOylation. Importantly, the motif is conserved between Rep proteins from different geminiviruses. Functionally, the motif was critical for Rep’s interaction with components of the SUMO machinery, for viral DNA replication, and for its ATPase activity. In particular, the third position of the motif was important for each of these activities. We thus uncover a hitherto undescribed mechanism on how geminiviruses recruit the SUMO machinery.

## INTRODUCTION

Circular Rep-encoding single-stranded (CRESS) DNA viruses form a unique group of viruses that use rolling-circle replication (RCR) to copy their genomes (1–3). They have been found in almost all branches of the eukaryotic tree of life. The *Geminiviridae* family of viruses represents an important group of plant-infecting viruses that cause severe crop damage (4). Interestingly, CRESS-DNA viruses have only one protein in common, i.e., replication initiator protein (Rep, C1), that orchestrates RCR (5, 6). To this end, these viruses need to recruit host factors, including DNA polymerases, to form viral replisomes with a partially unresolved but critical role for Rep (7–11). At the same time, Rep manipulates cellular processes by directing protein modifications such as ubiquitin or small ubiquitin-like modifier (SUMO) conjugation (SUMOylation) onto host proteins (12–14). It is well established that many viruses manipulate host SUMOylation to suppress antiviral responses or to enhance virulence (15). Yet, how and why Rep recruits and directs SUMOylation via one or more target proteins remains enigmatic.

Three protein domains have been identified in Rep. Its N-terminal domain, called the HUH (His-hydrophobic residue-His) endonuclease domain, nicks and joins the single-stranded viral genome at the origin of replication using an active-site tyrosine that forms a 5′-phosphotyrosine bond with the nicked DNA (16). The central domain is involved in Rep oligomerization (17–19), whereas the C-terminal domain shares homology with the adenosine triphosphate (ATP)ase helicase superfamily 3 (SF3 helicase) and is composed of a canonical Walker A and B motifs. This domain is presumed to act as a replicative helicase during RCR elongation (19–24). It was reported earlier that Rep from different geminiviruses is also able to reprogram the host cell cycle, thereby reactivating DNA replication in terminally differentiated plant cells (25, 26). In this process, Rep interacts with a multitude of host proteins that are implicated in the cell cycle (notably the DNA replication fork) and in DNA repair. For example, Rep was found to interact with transcriptional regulators such as retinoblastoma-related protein (RBR) (27, 28), as well as with proliferating cell nuclear antigen (PCNA), replication factor C (RFC), replication protein A32 (RPA32), and minichromosome maintenance protein 2 (MCM2) (29–32).

The general notion is that by the process of SUMOylation, the entire protein complexes become decorated with SUMO proteins attached to different sites, rather than that one substrate is modified at a single site by a single SUMO protein (33). In this way, SUMOylation modulates a wide range of nuclear processes, including the cell cycle, DNA replication, DNA damage repair, RNA processing, and gene expression (34). SUMO attachment involves a cascade of enzymatic reactions that starts with (i) precursor maturation, followed by (ii) SUMO activation by the SUMO E1-activating enzyme (SAE1/SAE2 dimer), and (iii) SUMO transfer to target proteins by the SUMO E2-conjugating enzyme 1 (SCE1) (35–39).

Rep connects DNA replication to the host’s SUMO modification machinery, modifying nuclear processes, such as cell cycle regulation and DNA repair (12, 14). Importantly, in the case of two geminiviruses, i.e., tomato yellow leaf curl virus (TYLCV; *Begomovirus coheni*) and tomato golden mosaic virus (TGMV; *B. solanum aureimusivi*), Rep was confirmed to interact with the SCE1 protein (12, 14, 40). Interestingly, the HUH domain of Rep^TGMV^ was found to interact with SCE1 (12). Furthermore, in the presence of Rep, SUMOylation of PCNA was impaired, both *in vitro* and *in vivo* (14). At least in yeast, loss of PCNA SUMOylation is known to cause an increase in homologous recombination (41), a process critical for geminivirus DNA replication (42). Two lysine residues in the HUH endonuclease domain of Rep^TGMV^ proved to be essential for SCE1 binding and viral replication/spread inside the host plant (40). However, the same lysines were not essential for the interaction between Rep^TYLCV^ and SCE1 (43). Instead, mutating these lysine residues plus one additional lysine resulted in cytosolic accumulation of Rep^TYLCV^ (43). Notwithstanding, Rep^TYLCV^ was found to interact with SCE1 in nuclear protein aggregates/foci called nuclear bodies (NBs), which possibly serve as hubs for interactions between viral and host proteins (43).

Here, we report how Rep^TYLCV^ interacts with Arabidopsis SUMO1 by revealing the existence of a hitherto unknown non-canonical SUMO-interacting motif (SIM) in the SF3 helicase domain. Our data imply that this SIM mediates interactions with both SUMO1 and SCE1. Mutation of this motif significantly impairs viral accumulation, indicating its functional importance. Since this SIM is positioned next to the Walker A motif, we further investigated its role in Rep’s ATPase activity. Our results demonstrate that the SIM not only promotes the interaction with proteins of the SUMO pathway, but it is also critical for ATPase activity and viral replication.

## RESULTS

### Rep from TYLCV interacts with SUMO1 and SCE1 via a non-canonical SIM in the SF3 helicase domain

**Fig 1:**
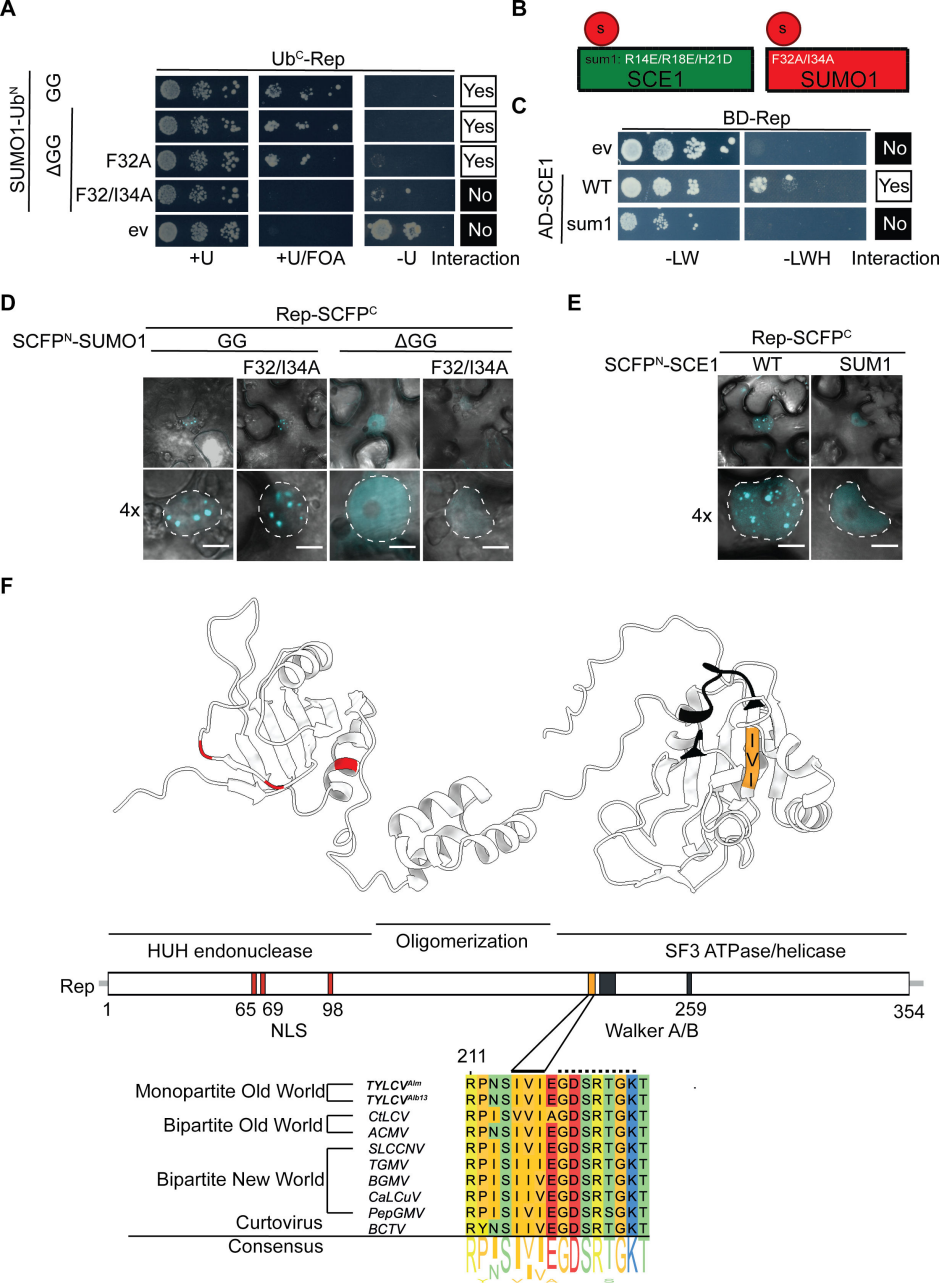
Rep interacts with SUMO1 and SCE1 via a predicted putative SUMO-interacting motif (SIM). (**A**) Split-ubiquitin Y2H assay between Rep (fused to C-terminal half of ubiquitin, Ub^C^) and different SUMO1 variants (Ub^N^). Positive interactions result in yeast growth on minimal medium (MM) supplemented with Uracil and FOA (+U/FOA) and no growth on MM lacking Uracil (−U). +U, positive control for normal growth. (**B**) Diagram depicting the SUMO1 and SCE1 variants used in the Y2H and BiFC assays in panels (**A**) and (**D**) and (**C**) and (**E**), respectively. (**C**) Gal4 Y2H interaction between Rep_Alb13_ and two SCE1 variants, i.e., WT protein and a variant mutated in the second SUMO binding site (sum1). −LWH, growth when bait and prey interact; −LW, positive control for normal growth. (**D, E**) Images depict a typical *N. benthamiana* epidermal cell (top), with a 4× zoom showing only the nucleus (bottom). Dotted lines delineate the nuclei; scale bar, 5 µm. (**F**) 3D model of Rep generated with AlphaFold2 showing the different domains and motifs. From left to right: HUH endonuclease, central oligomerization domain, and superfamily 3 ATPase/helicase domain. Colored residues depict the different Rep motifs, including the nuclear localization signal (NLS; red), SIM (orange), and Walker A and B motifs (black). Protein sequence alignment of the Rep SIM is shown from different geminiviruses. The shown sequence comprises the residues 211–226. Residues 215–217 of Rep_Alm_ and 217–219 of Rep_Alb13_ represent the SIM (*continuous line*), and residues 219–225 depict the Walker A motif (dashed line).

Previously, two Rep proteins (from TGMV and TYLCV) were found to interact with SCE1 from *Nicotiana benthamiana* and *Arabidopsis thaliana* (12, 14, 40, 43). As proteins involved in SUMOylation often interact with SUMO too in a non-covalent manner, we wondered whether (i) Rep^TYLCV^ (hereafter referred to simply as “Rep,” unless stated otherwise) also interacts with SUMO and (ii) whether this interaction involves a previously unknown SIM in Rep. To test this hypothesis, we first used the yeast two-hybrid (Y2H) split-ubiquitin system (SUS) (Fig. 1A). This system was chosen over the more commonly used GAL4-based Y2H system, as Arabidopsis SUMO1 (hereafter referred to as SUMO1) exhibited autoactivation when expressed as a bait protein (BD-SUMO) in the GAL4 system. Using the Y2H SUS, we found that Rep interacts with mature SUMO1 (GG), as well as with a conjugation-deficient variant (ΔGG) of SUMO1 that lacks the characteristic diGly motif at the C-terminus required for SUMOylation. This strongly suggests that a hitherto unknown SIM could be present in Rep. To further explore this possibility, we tested whether Rep could interact with a SUMO1 variant where two residues critical for SIM binding (F32/I34A) were mutated (Fig. 1B) (44). As expected, Rep failed to interact with this SUMO1^F32/I34A^ variant (Fig. 1A).

Earlier, it was reported that SCE1 can bind SUMO through two distinct mechanisms: (i) covalently via its catalytic pocket and (ii) non-covalently via a second binding site distal to its catalytic pocket (44, 45). Mutating this second site impairs SUMO binding. As the Rep-SCE1 interactions appear to be relatively weak, we wondered whether this non-covalent interaction could, in a cooperative manner, promote the Rep-SCE1 interaction. To test this, we employed the SCE1 variant R14E/R18E/H21D (hereafter SCE1^SUM1^), which lacks the ability to interact with wild-type (WT) SUMO1 (44). Using the GAL4 Y2H assay, we observed that Rep interacts with WT SCE1, but not with SCE1^SUM1^ (Fig. 1C). Thus, at least in yeast, the formation of a Rep-SCE1 complex depends (in part) on the ability of SCE1 to interact with SUMO1. Importantly, we anticipate that the yeast homologs of SUMO and SCE1 may serve as functional substitutes for the two plant proteins in this Y2H assay considering the strong conservation of the SUMO pathway across eukaryotes, which we did not investigate further.

Next, using the bimolecular fluorescence complementation (BiFC) assay, we investigated whether the ability of SUMO1 to bind the SIM also controls *in planta* the ability of Rep to interact with SUMO1. Previously, we had demonstrated that (i) the SUMO1-SCE1 protein complex tends to aggregate in large bodies in the nucleus, termed nuclear bodies (NBs), and that (ii) formation of these NBs depends on the recruitment of active SCE1 proteins to this complex (44). Similar to the SUMO1-SCE1 NBs, we now found that (i) Rep interacts with SUMO1 and SCE1 inside such NBs in this BiFC assay, and (ii) reconstitution of the fluorophore halves inside these NBs was suppressed when Rep was co-expressed with SUMO1^ΔGG+F32/I34A^ (Fig. 1D) or SCE1^SUM1^ (Fig. 1E). These data suggest that Rep interacts with SUMO1 and possibly SCE1 via a SIM in Rep.

Classically, SIMs are characterized by a stretch of at least three long-branched aliphatic residues, [VIL]-X-[VIL][VIL] (where “X” denotes any residue), flanked by a stretch of negatively charged residues (i.e., Glu, Asp, or phosphorylated residues) (46–50). To screen for candidate SIMs in Rep, the tool GPS-SUMO was used (https://sumo.biocuckoo.cn/https://sumo.biocuckoo.cn/). This yielded one non-canonical SIM motif in the C-terminal SF3 helicase domain consisting of a stretch of three long-branched aliphatic residues, i.e., I215, V216, and I217. This candidate SIM does not overlap with the coding sequence of the two known viral ORFs (C2 and C4) that overlap with Rep (C1) (51, 52). Yet, it is present in a β-strand adjacent to the Walker A motif of the Rep SF3 helicase domain (Fig. 1F). This means that the SIM is present in a different protein domain than the HUH endonuclease domain, which was previously reported to be important for the interaction between Rep^TGMV^ and SCE1 (40).

To assess whether this consensus motif is generally conserved across geminiviruses or even CRESS-DNA viruses, the degree of conservation of the motif was determined. This pan-genome comparison revealed that the candidate SIM is highly conserved in the Rep protein sequence of both mono- and bipartite begomoviruses (Fig. S1A) as well as CRESS-DNA viruses (Fig. S1B). In contrast, the same motif was detected in only 35% of the SF3 helicase sequences retrieved of more distant DNA viruses (Fig. S1C and D). To evaluate the structural impact of substituting the IVI motif for three alanines (AAA), structure predictions were performed using AlphaFold2 (AF2) (53) and AlphaFold3 (AF3) (54). Both AF2 and AF3 predicted the SF3 helicase domain of Rep with high confidence, based on the predicted local distance difference test (plDDT) scores (Fig. S2). No major structural differences were observed between WT Rep and Rep^sim^, with a root mean square deviation (RMSD) of 2.57 Å (Fig. S2B and C), suggesting that the IVI-to-AAA substitution does not induce a severe conformational change or negatively impact the SF3 helicase domain.

**Fig 2:**
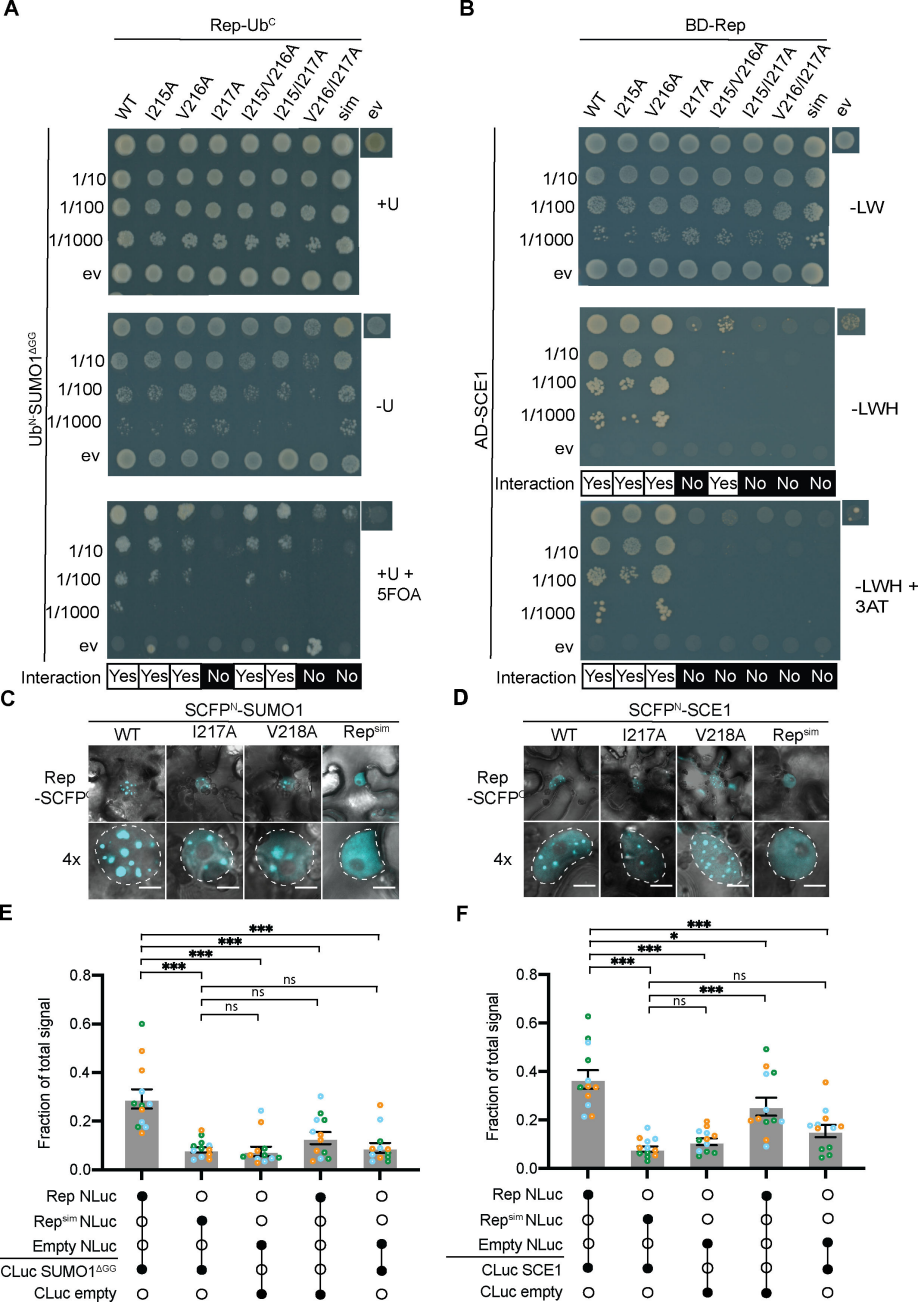
SIM is needed for Rep to interact with both SUMO1 and SCE1. (**A**) Split-ubiquitin yeast two-hybrid (Y2H) assay between Rep SIM mutants fused to C-terminal half of ubiquitin (Ub^C^) and SUMO1^DGG^ fused to Ub^N^. ev, empty vector control. Top to bottom: tenfold dilution series of the yeast cultures. Similar to the legend of Fig. 1A. (**B**) Gal4 Y2H assay between Rep SIM variants (GAL4 BD) and SCE1 (GAL4 AD). ev, empty vector control. Top to bottom: tenfold dilution series of the yeast cultures. Similar to the legend of Fig. 1C. (**C, D**) Bimolecular fluorescence complementation assay showing SUMO1 (**C**) and SCE1 (**D**) localization pattern in complex with Rep_Alb13_ and the Rep_Alb13_ SIM variants. Nuclei are outlined by a dashed white line; scale bars represent 5 µm. nls, nuclear localization-deficient variant (43). (**E**) Split-luciferase complementation assay between different Rep variants and SUMO1^DGG^. Each data point represents a technical replicate, while colors represent independent biological repeats. X-axis: filled circles represent co-expression of the indicated Rep/SCE1 constructs fused to either the N-terminus (NLuc) or C-terminus (CLuc) of the luciferase. ANOVA followed by a Dunnett multiple comparisons test was performed; ns, non-significant; *P < 0.05; ***P < 0.001; Error bar represents standard error of the mean (SEM) (n = 12). (**F**) Split-luciferase complementation assay between different Rep variants and SCE1. ANOVA followed by a Dunnett multiple comparisons test was performed. Other details as panel **E**.

To test that this motif serves as a *bona fide* SIM critical for the interaction of Rep with SUMO1 and SCE1, we then used a mutation strategy and tested the interactions of the mutants in the Y2H assay. To this end, each of the three aliphatic residues of the SIM was mutated to alanine, i.e., alone as single residue mutations and in all possible combinations (double and triple mutants). In the case of SUMO1, we found that the Rep single and double mutants I215A, V216A, I215/V216A, and I215/217A were still capable of interacting with SUMO1^ΔGG^, while the variants I217A, V216/I217A, and the triple mutant I215/V216/I217A (hereafter Rep^sim^) did not (Fig. 2C; Fig. S4A). For SCE1, we noticed that the double mutant I215/V216A and all Rep mutants that included the I217A mutation failed to interact (Fig. 2D; Fig. S4B). However, we cannot exclude that the absence of a protein interaction in the Y2H assay was caused by reduced protein expression of the fusion products in yeast.

To confirm our Y2H findings, we used two *in planta* assays: the BiFC and split-luciferase protein-protein interaction assays (Fig. 2C through 2F). In the case of the BiFC assays (Fig. 2C and D), we employed the Rep protein from TYLCV isolate “Alb13,” which is 93% similar to Rep from the isolate “Alm”; the latter Rep was used throughout the remainder of this study. Regardless of the isolate used, the results were consistent. For clarity, the respective residue positions of the SIM of the two variants Rep_Alb13_ and Rep_Alm_ are given in Table S1 (as there is a single residue shift in the SIM position). We found that the WT Rep and the single mutants localized in NBs with SUMO1 (Fig. 2C) and SCE1 (Fig. 2D). However, the Rep^sim^ triple mutant failed to aggregate inside NBs when co-expressed with SUMO1 (Fig. 2C; Fig. S3A) or SCE1 (Fig. 2D; Fig. S3B), which corroborates the Y2H data.

To assess whether the two BiFC protein pairs Rep-SCE1 and Rep-SUMO1 co-localize inside the same NBs, we also performed a multicolor BiFC experiment (Fig. S4). We observed that the reconstituted fluorescence signal of Rep-SCFP^C^/Venus^N^-SUMO1 overlapped nearly completely with the fluorescence signal of Rep-SCFP^C^/SCFP^N^-SCE1 inside NBs (Fig. S4A, *R* = 0.936). To further confirm specificity, we also examined whether the reconstituted BiFC signal of Rep-SUMO overlapped with the signal of SCE1 tagged with RFP (RFP-SCE1) inside NBs (Fig. S4B). This was indeed the case (*R* = 0.919). Thus, these findings indicate that Rep is recruited by SUMO1 and SCE1 inside the same NBs, resulting in the apparent formation of a ternary complex. Moreover, these NBs are likely formed as a result of SUMO conjugation activity, as previously highlighted by Mazur et al. (44). In an earlier study, we used the Rep protein from TYLCV_Alb13_ (43), an isolate distinct from the model strain TYLCV_Alm_ used throughout the rest of this study. In the following experiments, we used the TYLCV_Alm_ Rep to align with the commonly used model strain.

To independently validate the role of the Rep SIM, we examined its interaction with SUMO1 and SCE1 using a second assay, the split-luciferase assay (55). In line with our hypothesis, we found that expression of Rep with SUMO1^ΔGG^ or SCE1 resulted in enhanced luciferase activity compared to the negative controls (Fig. 2E and F). In contrast, expression of Rep^sim^ with SUMO1^ΔGG^ (Fig. 2E) or SCE1 (Fig. 2F) yielded a luciferase signal similar to or below the background signal observed for the negative controls, e.g., Rep  +  CLuc empty vector, indicative of a compromised interaction. When we tested the interaction of Rep^TGMV^ with SUMO1^ΔGG^ and SCE1 in this assay, we noticed that the interaction of Rep^TGMV^ is weaker than that of Rep^TYLCV^ with both SUMO1^ΔGG^ and SCE1 (Fig. S5A and B). When Rep^TGMV^-SCE1 was co-expressed, a relatively high background luminescence signal was observed for the pair Rep^TGMV^-NLuc/CLuc empty control (Fig. S5B). Nevertheless, we find that an intact SIM is required for Rep^TYLCV^ to interact with SUMO1^ΔGG^ in both yeast and *in planta* and that Rep^TGMV^ also interacts with SUMO1^ΔGG^ *in planta* in a SIM-dependent manner. However, the split-luciferase assay did not confirm the role of the Rep^TGMV^ SIM for the interaction with SCE1.

### Rep SIM is essential for viral DNA replication activity in *N. benthamiana*

**Fig 3:**
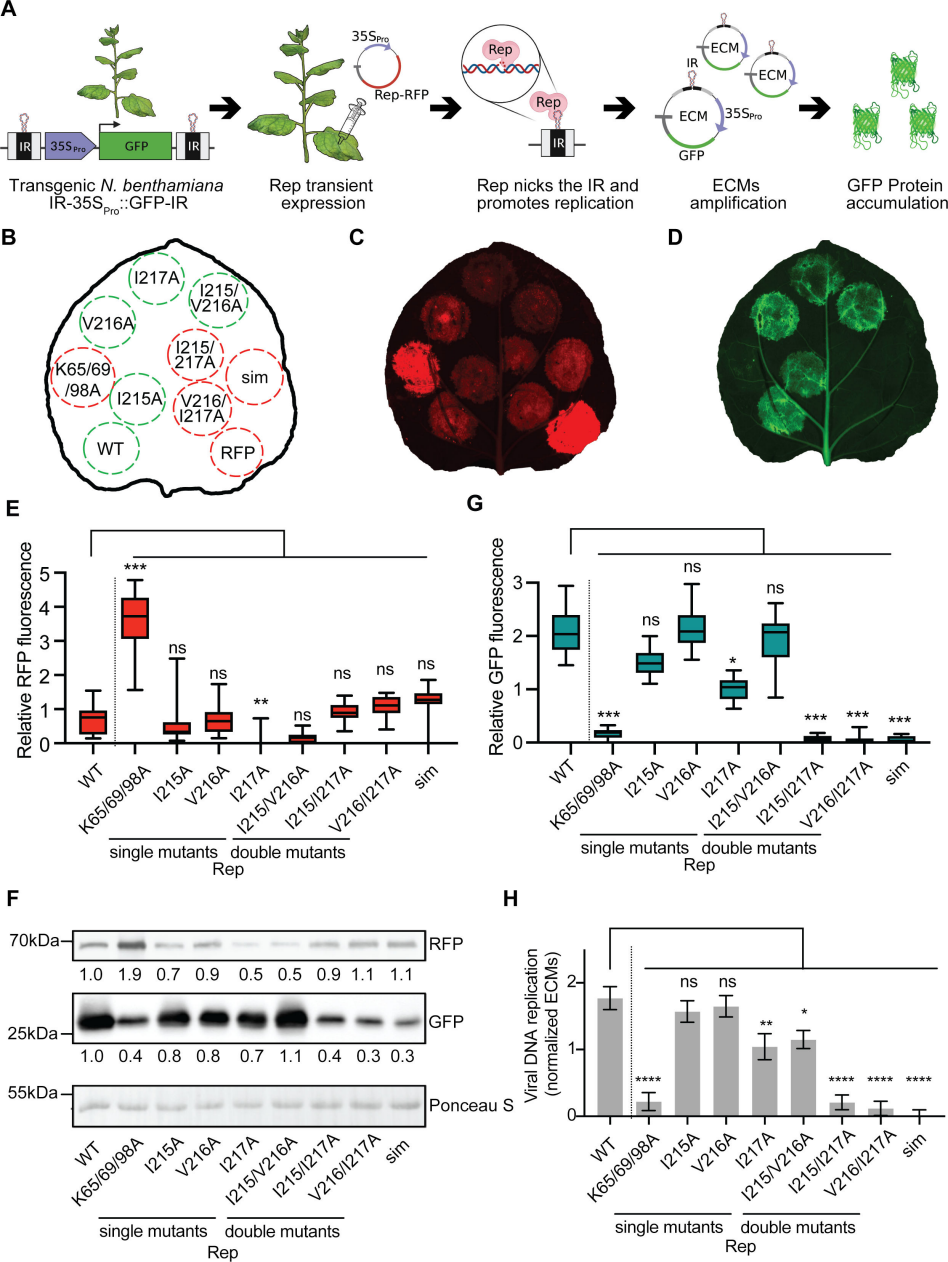
SIM is important for Rep-mediated viral DNA replication in a virus-free system (**A**) A reporter system to mimic and visualize geminiviral DNA replication in *N. benthamiana* plants in the absence of the intact virus. Transgenic *N. benthamiana* expressing *IR-35S_Pro_::GFP-IR* reporter is agroinfiltrated with a vector to deliver the viral Rep-RFP fusion protein. Upon transient expression, Rep-RFP nicks the DNA nicking at the IR motif and promotes RCR of the nicked DNA, resulting in the formation of circular ECMs. Each ECM contains a single *35Spro::GFP* cassette resulting in massive GFP accumulation. (**B**) Schematic depicting the agroinfiltrated Rep constructs. Clockwise from the bottom left: WT; K65/69/98A (nls), replication-deficient negative control impaired in nuclear localization (43); different SIM mutants, single (I215A, ...), double (I215/V216A, ...), triple residue (sim) mutants, and RFP as negative control. Green and red circles depict replication-competent and replication-deficient Rep variants, respectively. (**C, D**) Fluorescence images showing accumulation of Rep-RFP and GFP as a proxy for viral replication. (**B**) The layout of the infiltrated constructs. (**E**) RFP quantification of fluorescence signal in infiltrated leaf areas. Total fluorescence was normalized per leaf. Kruskal-Wallis test with Dunn’s correction was performed on the pooled data from each replicate. ns, non-significant; *P < 0.05; **P < 0.01; ***P < 0.001. Error bar represents SEM (n = 14). (**F**) Immunoblot showing both Rep-RFP and GFP levels. Ratios are compared to signal intensity of WT Rep. To demonstrate equal protein loading and normalize the blot signal values, membranes were stained with Ponceau S. (**G**) Similar to panel E, except that GFP was quantified. Other details are the same as in panel **E**. (**H**) Quantification of the ECMs using real-time PCR. ANOVA followed by Dunnett’s multiple comparison test was performed; *P < 0.05; **P < 0.01; ***P < 0.001; ****P < 0.0001. Error bar represents SEM (*n* = 3).

Next, we assessed whether the Rep SIM was critical for Rep viral DNA replication. To this end, we used a virus-free plant reporter system that mimics viral DNA replication (*2IR-GFP N. benthamiana*; Fig. 3A) (56). In this system, transient expression of *Rep* (in absence of any other viral protein) is sufficient to promote RCR of a *2IR-GFP* transgene cassette, thereby forming circular ssDNA extrachromosomal molecules (ECMs) that comprise a *GFP* expression cassette (*35S_Pro_::GFP*) (56). A TYLCV infection in this plant will result in mass production of GFP in cells where the virus replicates. Importantly, this plant reporter line was originally selected for the fact the *GFP* transgene is partially silenced, and this silencing does not affect the latter expression of *GFP* from ECMs formed. Nevertheless, the line already displays some basal *GFP* expression, which is independent of *Rep* expression.

Using this reporter, we tested whether the different SIM mutations disrupt the ability of a RFP-tagged Rep protein (Rep-RFP) to mediate viral DNA replication upon transient expression *in planta* (Fig. 3B and C). As negative controls, we expressed (i) RFP alone and (ii) an RFP-tagged replication-deficient variant of Rep that no longer accumulates in the nucleus (Rep^nls^; K65/69/98A) (43). First, we assessed whether the Rep SIM variants accumulated *in planta* and properly localized to the nucleus (43, 55). Mutating the Rep SIM did not change the nuclear localization of three Rep-RFP fusion variants tested (Fig. S6A) (43, 57). We then observed the DNA replication activity of the Rep SIM variants using the *2IR-GFP* reporter (Fig. 3D). All the Rep-RFP fusions showed protein accumulation, but some exhibited only 50% of the level of the WT Rep protein (Fig. 3E and F) (43). When assessing DNA replication activity, we found that the Rep variants I215/217A, V216/I217A, and I215/V216/I217A each showed no clear DNA replication activity; their GFP signals were comparable to those of the two negative controls, whereas expression of WT Rep and the other Rep SIM variants resulted in increased GFP signals (Fig. 3D, F and G). Consistently, the ECM levels were also lower for these three Rep SIM variants compared to WT Rep, demonstrating that the cause was reduced DNA replication activity rather than enhanced *GFP* silencing or translational arrest (Fig. 3H).

To further exclude that low GFP levels correlated positively with low Rep-RFP levels, both the RFP and GFP protein levels were quantified using immunoblotting and normalized to WT Rep-RFP (Fig. 3F). Based on the RFP fluorescence signal, I215/217A, V216/I217A, and I215/V216/I217A each accumulated to protein levels similar to WT Rep (i.e., in the range of 0.90–1.10) (Fig. 3E and F). As previously observed (43), the Rep^nls^ variant K65/69/98A, which accumulates in the cytosol, showed increased protein levels compared to WT Rep, likely due to differences in the protein extraction efficiencies between the cytosol and nucleus (Fig. 3E and F; Fig. S6B). These results indicate that these three Rep SIM variants with reduced SUMO1- and SCE1-binding (I215/217A, V216/I217A, and I215/V216/I217A) also exhibit impaired DNA replication activity without affecting their expression pattern or protein stability. It thus appears that mutating residue I217 has a strong negative impact on Rep DNA replication activity in combination with mutating residue I215 or V216.

### Rep SIM is also required for TYLCV accumulation and viral spread in plants

Having confirmed that an intact SIM is important for Rep DNA replication activity outside the context of the virus, we introduced the SIM mutations in a TYLCV infectious clone to exclude whether other viral ORFs can functionally compensate for the SIM mutations—both locally and in a systemic infection. To this end, a TYLCV infectious clone was generated as previously reported (58). In parallel, three mutant clones were generated, each containing different alanine substitutions in the Rep SIM coding sequence, while leaving the other known protein-coding viral ORFs intact (59). Based on the residual DNA replication activity of Rep I215/V216A, we hypothesized that the corresponding TYLCV clone would still be replication active, while the viral clones encoding the Rep variants I215/217A and I215/V216/217A (TYLCV^sim^) were expected to display strongly compromised replication activity. The TYLCV clones were each delivered into the *2IR-GFP N. benthamiana* plants using agroinfiltration, and the GFP levels were quantified in the infiltration zone (Fig. S7A and B). Wild-type TYLCV and TYLCV Rep I215/V216A caused, respectively, strong and weak GFP fluorescence in the infiltration site (Fig. S7B and C). The viral clones encoding Rep I215/217A and Rep^sim^ instead yielded a GFP fluorescence signal comparable to that of the mock control. Quantification of viral DNA levels in these experiments using real-time PCR yielded similar results (Fig. S7D). The clone encoding Rep I215/V216A showed near-WT activity in viral DNA replication. As expected, the TYLCV clones encoding Rep I215/217A and Rep^sim^ showed a clear reduction in DNA replication activity, with viral DNA titers being 1–2 magnitudes lower than those of WT TYLCV.

**Fig 4:**
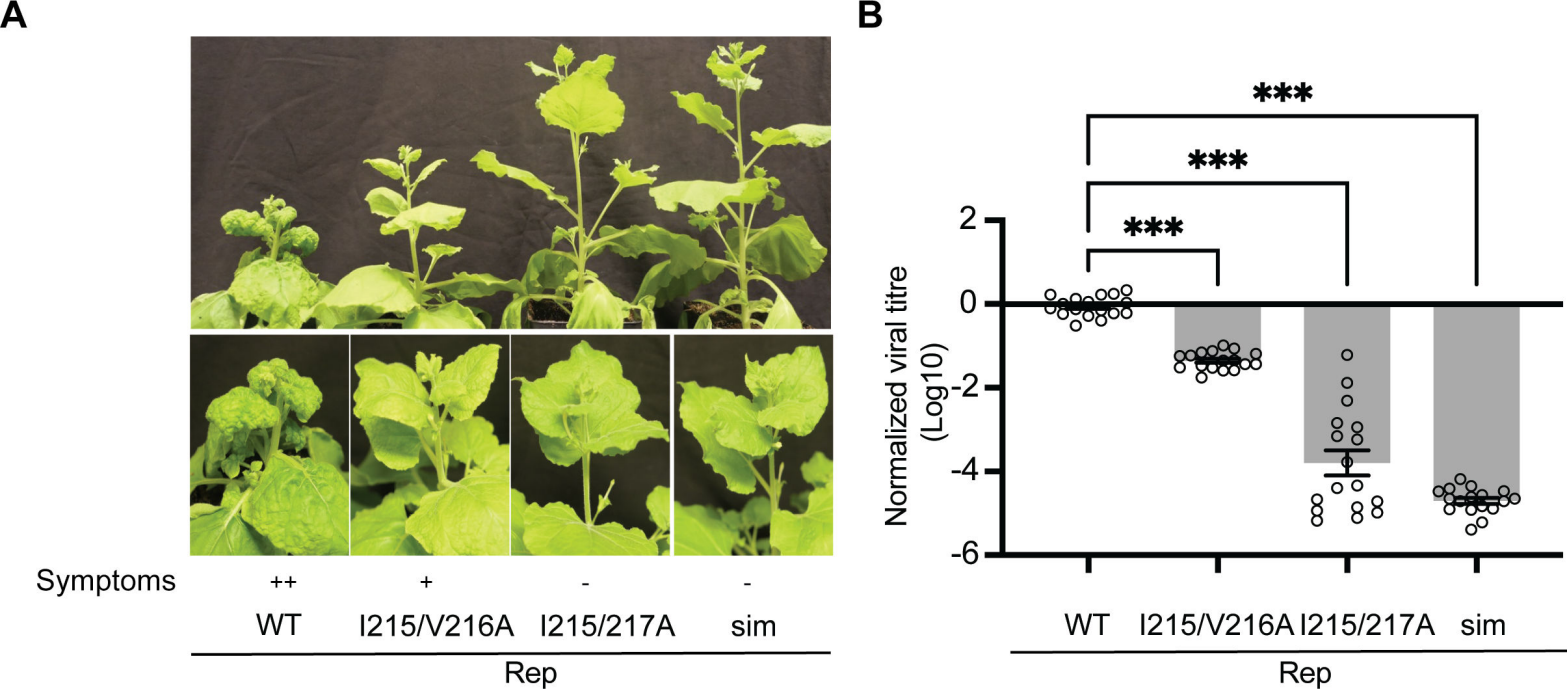
The Rep SIM is crucial for TYLCV to induce disease symptoms and enable viral genome replication in *N. benthamiana*. (**A**) *2IR-GFP N. benthamiana* plants infected with different TYLCV infectious clones encoding either WT Rep or Rep SIM variants (I215/V126A, I215/I127A, I215/V126A/I127A = sim). TYLCV disease symptoms are indicated: ++, severe symptoms; +, mild symptoms; −, no symptoms. Photos were taken three weeks post-inoculation. (**B**) Quantification of viral titers using real-time PCR on the extracted DNA from the apical leaves. Levels were normalized to the viral titers by the WT TYLCV (Log_10_ = 0). ANOVA followed by Dunnett’s multiple comparison test was performed; ***P < 0.0001. Error bar represents SEM (n = 17).

To assess whether viral replication was affected systemically, we monitored the infection 28 days post-agroinfiltration of *2IR-GFP N. benthamiana*. The WT TYLCV clone induced clear disease symptoms (including leaf curling, leaf puckering, and stunted plant growth) indicative of a systemic infection (Fig. 4A). As expected based on the former experiment, the TYLCV clone encoding Rep I215/V216A caused disease symptoms but to a lesser extent. In contrast, the TYLCV infectious clones encoding Rep I215/217A and Rep^sim^ failed to induce disease symptoms, thereby corroborating our hypothesis that the SIM of the Rep^TYLCV^ is critical for viral infectivity. Real-time PCR analysis confirmed this phenotypic data (Fig. 4B), showing that viral DNA titers were low for the TYLCV clones encoding Rep I215/I217A and Rep^sim^ compared to the WT clone in systemically infected tissue. For the TYLCV clone encoding Rep I215/V216A, the viral titers were intermediate, approximately one order of magnitude lower than those observed for WT TYLCV.

The *2IR-GFP N. benthamiana* reporter line was previously selected for its low basal GFP fluorescence, indicative of partial silencing of the *GFP* transgene in the reporter cassette, and for its capacity to exhibit increased fluorescence upon activation of viral DNA replication (56). Increased GFP fluorescence is visible up to 14 days post-infiltration, while its silencing is re-established from 21 days post-infiltration (60). To indirectly assess viral replication, we imaged GFP fluorescence in the *2IR-GFP N. benthamiana* plants. Delivery of the WT TYLCV or TYLCV Rep I215/V216A clones led to reduced GFP fluorescence, consistent with active viral replication and subsequent silencing of the *2IR-GFP* construct. In contrast, TYLCV Rep I215/217A and TYLCV^sim^ maintained their basal GFP fluorescence, consistent with an impaired replication capacity and failure to promote further GFP silencing (Fig. S7E). To exclude the possibility of a spontaneous reverting mutation that restores the mutated SIM sequence back to the original sequence and thereby enables viral spread, Sanger sequencing was performed on viral DNA extracted from systemic tissue. No clear evidence was seen in the Sanger reads that the mutated SIM region had undergone a reversion mutation in the viral genome in the systemically infected tissue (Fig. S7F), thus confirming that the lowered levels of viral replication, disease symptoms, and viral spread could be solely attributed to the SIM mutations introduced in the *Rep* ORF. These findings collectively demonstrate that the Rep SIM is critical for viral replication and systemic spread in the context of the whole TYLCV virus.

### Mutating the SIM impairs Rep ATPase activity

**Fig 5:**
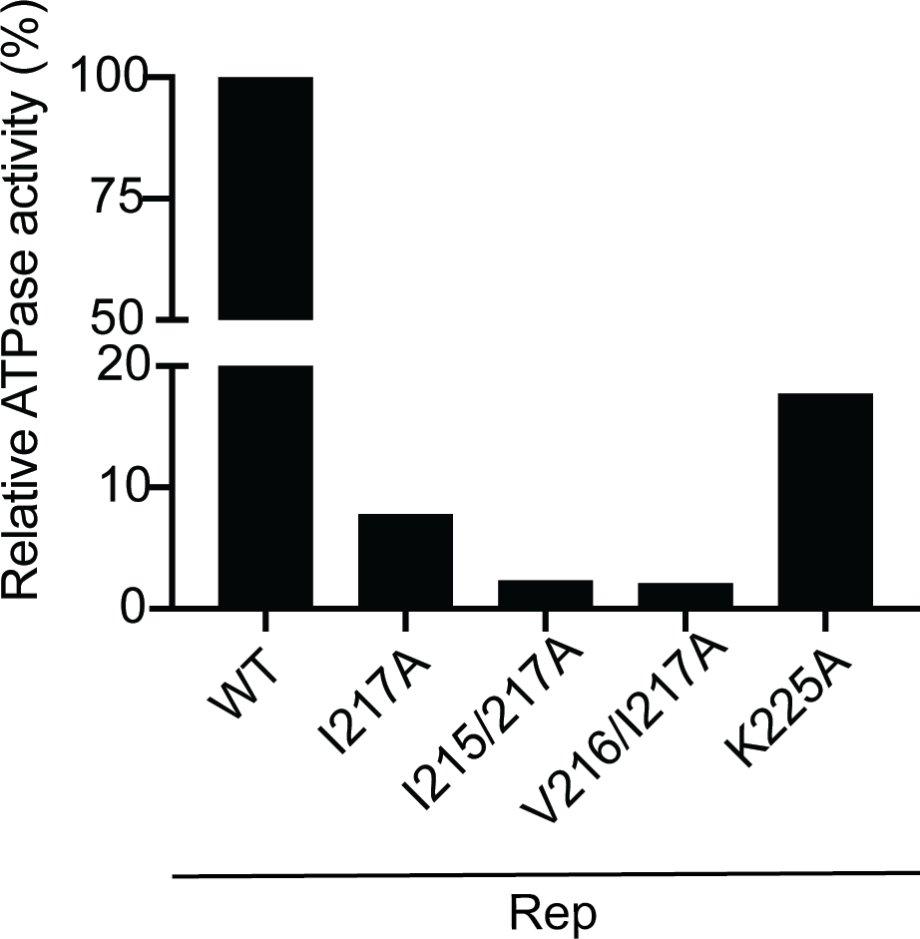
An intact SIM is needed for full Rep ATPase activity. Relative ATPase activity in purified protein fractions corresponding to WT Rep (normalized to 100% activity) versus the indicated Rep SIM mutants on the x-axis. All recombinant proteins were expressed in *E. coli*. K225A variant: known Rep mutant with lowered ATPase activity (negative control, see reference 20).

Due to the proximity of the SIM to the Walker A motif, mutating the SIM might also affect Rep ATPase and helicase activity (20) (Fig. 1F). It has been earlier shown that substituting K225 in the SF3 helicase domain of Rep^TYLCV^ for an alanine strongly impairs its ATPase activity (20). To investigate this possibility, we expressed and purified several Rep variants and tested their ATPase activity. Specifically, we focused on the I217A mutations, considering the involvement of this residue in both the SUMO1/SCE1 interaction and viral accumulation (Fig. 2 and 4). The WT Rep protein purified exhibited clearly more ATPase activity than the three SIM mutants tested, i.e., less than 10% of the activity of WT Rep. Notably, Rep I217A protein sample exhibited markedly reduced ATPase activity, even lower than that of the negative control, Rep K225A, a mutation previously reported to abolish ATPase activity (20) (Fig. 5). Given the residual activity observed for Rep K225A, we hypothesized that the residual ATPase signal in this negative control could originate from co-purifying *E. coli* chaperones with ATPase activity, such as Heat Shock Protein HSP70 (61), rather than from Rep protein itself (62). Nevertheless, these findings indicate that the I217A mutation already reduced Rep ATPase activity, and this activity was even lower for the Rep double mutations I215/216A and V216/217A. Collectively, these results underscore again the key role of the I217 residue in the Rep SIM, not only for the SUMO1/SCE1 interactions, but also for viral replication and ATPase activity.

## DISCUSSION

CRESS-DNA viruses all encode a single conserved protein, Rep, which orchestrates viral DNA replication. Previous studies reported that Rep^TGMV^ interacts with the SUMO E2 conjugating enzyme SCE1 via its N-terminal HUH endonuclease domain (12, 14, 40). Here, we established that the SUMO protein itself is apparently involved in this interaction between Rep^TYLCV^ and SCE1. Specifically, we provided evidence for the existence of a not earlier studied non-canonical SIM in the SF3 helicase domain of this Rep. This SIM was not only required for Rep^TYLCV^ to interact with SUMO1, but also with SCE1. Using a virus-free reporter system for viral DNA replication activity (*2IR-GFP N. benthamiana*) (56), we demonstrated that replacing two residues in this SIM for alanines, specifically the third residue along with either the first or second residue in the motif, was sufficient to apparently strongly inhibit RCR by Rep. Likewise, mutating the first and third positions combined was sufficient to prevent viral replication, systemic spread, and disease symptom development of an otherwise functional infectious clone of TYLCV, demonstrating that none of the other viral transcripts or translated proteins were able to compensate for the introduced mutations in the Rep ORF. The overall significance of this SIM is underscored by its apparent conservation across different geminivirus Rep proteins, including both monopartite and bipartite begomoviruses (Fig. 1F), suggesting a conserved role in the viral infection cycle. Notably, while the SIM is conserved in geminiviruses and CRESS-DNA viruses that employ RCR, the motif was not strictly conserved in other SF3 helicase domains in unrelated DNA viruses, e.g., cottontail rabbit papillomavirus or bovine parvovirus 1 (Fig. S1). This reduced conservation suggests that the SIM is not essential for ATPase/helicase activity *per se*, unlike the Walker A motif that was strictly conserved across all the protein sequences analyzed (Fig. S1).

The requirement for an intact SIM for Rep^TYLCV^ to mediate the interaction with both SUMO1 and SCE1 was demonstrated using the Y2H assay and confirmed *in planta* through two independent methods: BiFC and the split-luciferase. It was previously reported that *Rep* overexpression did not result in a global inhibition of the SUMO conjugate levels *in planta* (40). Here, we make a related observation that, at least in the BiFC system, the protein pair Rep-SCE1 resides in NBs, similar to the SUMO1-SCE1 protein pair. As the formation of SUMO1-SCE1 NBs strictly depends on the presence of SCE1 SUMO conjugation activity in these bodies (44), the formation of these Rep-SUMO/SCE1 NBs corroborates again that the viral role of Rep is not to suppress global SCE1 enzyme activity (Fig. 2C and D), but more likely that it has a role in stimulating or repressing SUMOylation of one or more specific host proteins, as shown in the latter case for tomato PCNA (14).

Previous studies have reported that Rep^TGMV^ interacts with SCE1 via its N-terminal HUH endonuclease domain, with lysine mutations in this domain disrupting this interaction (12, 14, 40). Here, we identified a novel SIM present in the SF3 helicase domain of Rep^TYLCV^ and Rep^TGMV^ that is important for both proteins to interact in a non-covalent manner with a conjugation-deficient form (ΔGG) of SUMO1. However, in the case of the Rep^TGMV^-SCE1 protein pair, a relatively high background signal was observed in our split-luciferase assay (Fig. S4D), which prevents us from drawing firm conclusions on the role of the SIM in the case of the Rep^TGMV^-SCE1 interaction, while it was needed for the Rep^TYLCV^-SCE1 interaction. These findings do, nevertheless, support a conserved function for the SIM across geminiviruses, including mono- and bipartite begomoviruses. Our data also corroborate the earlier proposed notion by Maio et al. (43) that the mechanism that stabilizes the SCE1-Rep interaction likely differs between Rep^TYLCV^ and Rep^TGMV^.

We propose that, in the case of Rep^TYLCV^, a ternary complex is formed between Rep^TYLCV^, SCE1, and SUMO1, and this ternary interaction allows what would otherwise be a weak interaction to be detected in our assays. In this model, no single interaction predominates; rather, all three components contribute to the formation and stability of the ternary complex. This cooperative binding may alleviate competition between SUMO1 and SCE1. Notably, the Rep residue I217 appears to have a central role in coordinating this interaction between Rep^TYLCV^ and SUMO1/SCE1. Most other single and double residue mutations in the SIM gave a Rep variant that still interacted to some extent with SUMO1,^ΔGG^ except for Rep I217A, V216/I217A, and the triple mutant I215/V216/I217A (Fig. 2A). In the case of the interaction between Rep and SCE1, the double mutant I215/V216A, as well as any mutant carrying the I217A substitution, disrupted this interaction (Fig. 2B). The I217A mutation led to a more pronounced reduction in viral DNA accumulation (quantified using both GFP expression levels from ECMs and accumulation of viral DNA) compared to the single I215 or V216 single residue mutations (Fig. 3F). These findings thus add a novel function to the SF3 helicase domain of Rep, that is, SUMO binding.

Finally, we investigated the impact of the position of the SIM adjacent to the Walker A and B motifs within the SF3 helicase domain to determine whether SIM mutations negatively impact Rep ATPase activity. Indeed, mutating the SIM impaired Rep ATPase activity, providing a mechanistic explanation for the loss of RCR activity, as ATP hydrolysis is essential for Rep helicase function (63). In general, Walker A motifs are located on a flexible loop (also known as the “phosphate-binding loop” or in short “P-loop”) preceded by a β-strand and followed by an α-helix. Notably, previous studies have shown that a conserved stretch of hydrophobic residues is located on the β-strand upstream of the Walker A (P-loop) motif and identified the presence of hydrophobic residues at distinct positions on the subsequent α-helix (64). Those hydrophobic residues possibly interact with each other and thereby stabilize the arrangement of the two secondary structure elements relative to each other, which might in turn facilitate nucleotide (e.g., ATP) binding by the P-loop. By substituting isoleucines and valines in Rep that likely coincide with the hydrophobic stretch in the β-strand preceding the P-loop (Fig. S1) for alanines, the overall hydrophobic character of the motif was preserved. However, the smaller size of the alanine side chain compared to valine and isoleucine may have an effect on the proximity/angular arrangement of the two secondary structure motifs flanking the P-loop. Structural predictions with AlphaFold3 of Rep^sim^ suggested that the introduction of the alanines did not appear to negatively impact the integrity of the Walker A (P-loop motif) of the ATPase domain and resulted in comparable plDDT confidence values for this protein domain with respect to WT Rep (Fig. S2). Yet, these latter protein models should be taken with caution as AlphaFold was not designed to capture subtle effects of one or a few amino acid changes (65). To conclude, we find a three-residue motif that contributes to both the SUMO1/SCE1 interaction and viral accumulation. Whether this SIM is also involved in the suppression of PCNA SUMOylation by Rep remains an open question (14). While our findings advance our understanding of the Rep interaction with the SUMO machinery, further studies are needed to elucidate the specific mechanisms by which the SIM may modulate PCNA activity.

## MATERIALS AND METHODS

### General methods and cloning

All molecular techniques were performed using standard methods (66). *Escherichia coli* strain DH5α was used to clone gene fragments. All primers and plasmids used are described in the Tables S2 and S3, respectively. The different fragments were PCR-amplified using Phusion DNA polymerase (Thermo Fisher), and the amplicons obtained were introduced in pENTR207 or pENTR221 (Thermo Fisher) using Gateway BP Clonase II (Thermo Fisher). Transfer of the inserts to destination vectors was done using Gateway LR Clonase II (Thermo Fisher). Point mutations were introduced using QuikChange site-directed mutagenesis. All inserts were confirmed by DNA sequencing. The order of the different protein fusions (i.e., tag-REP or REP-tag) is indicated in the figures. Rep ORF from the TYLCV isolate “Almeria” was used in this study, unless mentioned otherwise.

### Yeast two-hybrid assays

For the GAL4 Y2H assay, all gene fragments were cloned into Gateway-compatible variants of pGADT7 and pGBKT7 (Clontech) (59). Resulting plasmids were introduced into *Saccharomyces cerevisiae* strain PJ69-4α (67) using a standard lithium acetate/single-stranded DNA/polyethylene glycol 3350 transformation protocol (68). Transformed colonies were selected on yeast minimal medium (MM) lacking the amino acids Leu and Trp. To select for protein-protein interactions (PPIs), three independent transformants were randomly picked. After resuspension in 100 µL sterile double-distilled water, a tenfold serial dilution was spotted on MM agar plates lacking Leu, Trp, and His (*–*LWH) supplemented with 1 mM 3-amino-1,2,4-triazole (3AT). The plates were then incubated at 30°C for 3 days prior to scoring yeast growth.

The Gateway-ready plasmids for the split-ubiquitin Y2H system, pMET/pCub (57), were transformed into *S. cerevisiae* JD53. Selection of transformants and positive PPIs was performed as described (69). Briefly, transformed colonies were selected on yeast MM lacking His and Trp (–HW). To select for PPIs, three independent transformants were resuspended, and a tenfold serial dilution was spotted (similar to GAL4 assay) on MM agar plates supplemented with 0.1 mM CuSO_4_ and the appropriate amino acids. To increase selection specificity*,* 1 mg/mL 5-FOA (5-fluoroorotic acid, Sigma) was added to the selection plates. Plates were incubated at 30°C for 4 days prior to scoring.

### Transient expression of proteins in *N. benthamiana* using agroinfiltration

All binary constructs were transformed in *Agrobacterium tumefaciens* (*Rhizobium radiobacter*) strain GV3101 (70) by electroporation (25 µF, 400 Ω, 1.25 kV/mm). Single colonies were grown overnight to an optical density at 600 nm (OD_600_) of 0.8–1.5 in low salt LB broth (1% [wt/vol] tryptone, 0.5% [wt/vol] yeast extract, 0.25% [wt/vol] NaCl, pH 7.0). Bacterial cells were collected by centrifugation (3,000 × *g* for 5 min), washed, and resuspended in leaf infiltration medium (1 × MS [Murashige and Skoog] salts, 10 mM MES pH 5.6, 2% [wt/vol] sucrose, 200 µM acetosyringone). *A. tumefaciens* carrying the pBIN61 binary vector to express the P19 silencing suppressor (referred to as pBIN61:P19) from tomato bushy shunt virus (TBSV, *Tombusvirus lycopersici)* (71) was added to every experimental sample.

### Bimolecular fluorescence complementation (BiFC) assay

*Rep* from the TYLCV isolate “Alb13” (*Rep*_Alb13_), the Arabidopsis *SUMO1* (gene ID: AT4G26840), and *SCE1* (AT3G57870) coding sequence were cloned in the vectors pDEST-GWSCYCE to express fusion protein with the C-terminal half of S(CFP)3A (residues 156–239; referred to as SCFPC) or pDEST-SCYNEGW (for protein fusions with the N-terminal half of S(CFP)3A residues 1-173; SCFPN) (72). Four-week-old *N. benthamiana* leaves were syringe-infiltrated with *A. tumefaciens* suspensions at a final OD_600_ of 1.0 when a single construct was delivered. When two cultures were co-infiltrated for BiFC analysis, the cultures were mixed at a ratio of 1:1 to a final OD_600_ of 1.0. *A. tumefaciens* carrying pBIN61:P19 was added to every experimental sample at a final OD_600_ of 0.5. Three days post-infiltration, *N. benthamiana* leaf material was collected to analyze the expression of the infiltrated constructs. SCFP fluorescence was detected using an excitation wavelength of 458 nm (argon laser), primary beam-splitting mirrors 458/514 nm, secondary beam splitter 515 nm, and band filter BP 470–500 nm.

### *In planta* protein localization

*Rep*_Alb13_ was cloned in the plasmid pGWB654, which carries a C-terminal monomeric red fluorescent protein (mRFP) tag (73). The expression and detection were the same procedure as for the BiFC assay.

### Confocal microscopy and image analysis

A confocal laser scanning microscope (Zeiss LSM510) was used to capture fluorescent images using a Zeiss c-Apochromat 40 × 1.2 water-immersion Korr objective. Fluorescence was detected using the following beam and filter settings: GFP excitation at 488 nm (argon laser), primary beam-splitting mirrors 405/488, secondary beam splitter 490 nm, band filter BP 505–550 nm; RFP excitation at 543 nm (helium-neon laser), primary beam-splitting mirrors 488/543 nm, secondary beam splitter 635 nm, and band filter LP 585–615 nm. For all observations, the pinhole was set at 1 Airy unit. The *Rep*_Alb13_ variant was used for the confocal work. For every experimental condition/treatment, at least three independent leaves were examined, and one representative image is shown (at least 50 nuclei were examined per sample). For each image analysis, absolute intensity values were used (Raw16.tif format). Images were analyzed and processed using ImageJ Fiji 1.0v (73, 74). Counting of the nuclear bodies was performed using pictures of 30 nuclei per treatment.

### Rep structure prediction

The 3D protein model of Rep (Accession: AJ489258.1) was generated using AlphaFold2 (53) and visualized with ChimeraX−1.8 (75, 76) or AlphaFold3 (AF3) (54).

### CRESS-DNA Rep alignment

The reference strain of each geminivirus genus was retrieved from Viralzone (https://viralzone.expasy.org/) and was used for the SIM geminivirus sequence consensus. In total, we retrieved 15 protein sequences. We searched the UniProtKB database for CRESS-DNA viruses using the search terms “Rep AND (taxonomy_id:2731342 (Monodnaviria)) AND (go:0004386 (helicase activity)).” In total, we retrieved 49 protein sequences. Additionally, we searched the UniProtKB database for SIM helicase superfamily 3 viruses using the search terms “Helicase, superfamily 3, DNA virus” (IPR014015). In total, we retrieved 144 protein sequences. To determine the percentage of sequences containing SIMs, the following consensus sequence was used: ψ–X₀–₂–ψ–ψ, where ψ = [V, I, L] and X₀–₂ represents any amino acid occupying 0 to 2 positions. UniProtKB and viralzone.expasy.org were consulted the 30th of October 2024, and only reviewed sequences were retrieved. The consensus logo was generated using Weblogo (https://weblogo.berkeley.edu/logo.cgi) on the 20 residues flanking the studied SIM.

### *In planta* DNA replication activity assay using heterologous expression of Rep

To assess Rep DNA replication in the absence of TYLCV, *N. benthamiana* expressing the *2IR-GFP* reporter cassette was used (43). Three- to five-week-old plants were co-infiltrated with (i) *A. tumefaciens* strain carrying pGWB654 plasmid encoding different *Rep-mRFP* variants (77) and (ii) *A. tumefaciens* carrying the pBIN61:P19 to suppress gene silencing (71). The bacterial cultures were mixed at a ratio of 2:1 to final OD_600_ values of 0.8 and 0.4, respectively, prior to infiltration. Four days post-infiltration, whole leaves were imaged for GFP and RFP fluorescence using a Chemidoc MP (BioRad) imager with the presettings “Alexa 488” and “Rhodamine,” respectively. To estimate the relative fluorescence intensity, three leaves of the same age from different plants were agroinfiltrated and imaged (*n* = 3). Mean fluorescence intensities per infiltrated area were calculated. To normalize fluorescence between leaves, the fluorescence is given as a proportion of the total intensity per leaf. Visualization and ANOVA tests were performed using Prism 9.0v (GraphPad).

### Quantification of extrachromosomal molecules using qPCR

To quantify the level of ECMs, leaf disk samples were snap-frozen in liquid nitrogen and stored at –80°C prior to tissue processing. Frozen tissue was ground to powder using a steel ball in a bead mill (TissueLyser II, Qiagen). Total DNA was extracted from approximately 30 mg of leaf tissue using a routine hexadecyltrimethylammonium bromide (CTAB) method (78). DNA concentrations were estimated using the absorbance at 260 nm on a Nanodrop (Thermo Fisher). In total, 250 ng of total DNA was used as template for a real-time PCR reaction (QuantStudio3, Thermo Fisher) using the Hot FIREPol EvaGreen qPCR kit according to the suppliers’ instructions (Solis Biodyne). The ECM signal was normalized using the *25S rRNA* (GenBank ID, KP824745.1) as an internal reference. The primers to detect the viral DNA were designed such that they only amplify ECMs and not the *2IR-GFP* genomic insert (Table S2). All cycle threshold (Ct) values were corrected for primer efficiencies. All expression data were analyzed following the standard workflow provided by qBASE+ (Biogazelle) using three independent biological replicates (*n* = 3). CNRQ was calculated using qBASE+. Data visualization and ANOVA tests were performed using Prism 9.0v (GraphPad).

### Protein extraction from plant tissue and immunoblotting

Total protein fraction was extracted using approximately 30 mg of frozen *N. benthamiana* leaf tissue. To this end, leaf material was snap-frozen and ground using plastic pestles, after which RIPA buffer (50 mM Tris-HCl pH 7.4, 150 mM NaCl, 1% [vol/vol] Triton X-100, 0.5% [wt/vol] deoxycholate, 0.1% [wt/vol] SDS, 5 mM DTT, 1 × cOmplete Protease Inhibitor Cocktail [Roche]) was added at a 4:1 (vol/wt) buffer-to-tissue ratio. Samples were thawed on ice and vortexed three times for 10 s. After incubating the homogenates prior on a rotating wheel for 1 h (25 rpm) at 4°C, they were centrifuged at 16,000 × *g* (4°C), and 80 µL of the supernatant was mixed with 2 × Laemmli buffer (100 mM Tris pH 6.8, 20% [wt/vol] glycerol, 4% [wt/vol] SDS, 100 mM DTT, 0.001% [wt/vol] Bromophenol blue) in 1:1 ratio. Total protein extract was denatured by heating at 96°C for 10 min. Upon protein denaturation, extracts were centrifuged at maximum speed (16,000 × *g*, ambient temperature) for 5 min, and the supernatant was separated on a 12% SDS-PAGE gel and subsequently transferred onto a PVDF membrane (Immobilon-P, Millipore). Immunodetection of the proteins was performed according to the standard protocols using the antibodies detailed in Table S4. For detecting chemiluminescence (ECL), a home-made solution was used (0.1 M Tris-HCl pH 8.5, 1.25 mM luminol [Sigma-Aldrich] in DMSO, 0.2 mM *p*-coumaric acid [Sigma-Aldrich] in DMSO, 0.01% [vol/vol] H_2_O_2_), and the signals were imaged using a Chemidoc MP (Bio-Rad) or a light-sensitive X-ray film (Fuji Super RX). Equal loading of the different protein extracts was confirmed by examining the Rubisco levels using Ponceau S staining of the membranes. The intensity of the Rubisco signal was also used to normalize the relative GFP and mRFP protein levels.

### Quantification of viral infections

Infectious clones are described in detail in Table S3. *Agrobacterium* carrying the pGreen TYLCV constructs was cultivated 24–30 h to saturation (OD_600_ 3.5–4) and then pelleted by centrifugation for 10 min (3,000 × *g*) before being resuspended in fresh low salt LB medium without antibiotics to a final OD_600_ of 7.5–8. Then, they were introduced into three-week-old *N. benthamiana* by injecting the suspension in an abaxial bud. Plants were maintained at 25°C with natural daylight supplemented to a 16-hour photoperiod with sodium lamps (Sylvania Gro-Lux). Three weeks post-inoculation, apical leaves were harvested near the apical shoot to isolate total DNA and quantify the relative viral accumulation of TYLCV coat protein in *N. benthamiana* by quantitative real-time PCR. To confirm that TYLCV mutants did not revert back to WT Rep protein sequence by spontaneous mutations and selection pressure, the virus samples were isolated and sequenced. One representative sequencing result of Rep (552–720 bp from the start codon) is shown (Fig. S7F). Normalization for viral DNA was performed using the ribosomal *RNA 25S* as internal reference. Primers used for real-time quantification are detailed in Table S2. Real-time PCR primers were designed to only amplify the circularized viral DNA.

### Split-luciferase complementation (SLUC) assay

*N. benthamiana* leaves were co-infiltrated with a mixture of *A. tumefaciens* harboring different pGWB402 (77) constructs (vectors used can be found in Table S3) and *A. tumefaciens* pBIN61:P19 with a final OD_600_ of 0.8 and 0.4 for both strains, respectively. *Rep*_Alb13_ variant was used for this SLUC. The infiltrated leaf areas were marked using a paint marker. Three days post-infiltration, agroinfiltrated leaves were brushed twice with D-luciferin buffer (235 mM D-luciferin [Duchefa Biochemie] dissolved in Milli-Q water, 0.02% [wt/vol] Silwet L-77 [Crompton Europe]). After 2 h dark incubation, the chemiluminescence signal was captured using a CCD imaging system (Princeton Instruments) set at −70°C. An exposure time of 15 min with 10 × 10 binning was used for images. Data acquisition was performed using the MetaVue program (X-Rite). Each data point consisted of at least three replicates, and four independent experiments were performed for each assay. As a negative control, unfused half of the luciferases were used. The log10 value of the mean signal (integrated density/area size), corrected for the leaf background signal and expressed as a proportion of total signal of all samples infiltrated in a single leaf, was used for further statistical tests. The data of each independent experiment were pooled for the ANOVA followed by Dunnett’s multiple comparison test in Prism v10.0 (GraphPad).

### Rep protein expression and purification

To express and purify recombinant Rep, the *E. coli* strain BL21-Gold (Agilent Technologies) harboring *Rep* in pGEX (with an N-terminal glutathione S-transferase (GST)-tag and PreScission-cleavage site) was used. Two-liter cultures were started with an overnight pre-culture to an OD_600_ of 0.1 and grown at 37°C and 200 rpm until reaching an OD_600_ of 0.7. Rep protein expression was induced using 0.5 mM IPTG, and the temperature was lowered to 30°C. After 2 h of induction, the bacterial cells were harvested at 5,000 × *g* for 15 min at room temperature, and the cell pellet was stored at −20°C prior to purification. The frozen pellet was thawed and resuspended in 30 mL of lysis buffer (50 mM Tris-HCl pH 7.5, 300 mM NaCl, 10% [wt/vol] glycerol, 2 mM MgCl_2_, 0.5 mM CaCl_2_, 2 mM DTT, 1% [wt/vol] Tween-20, 0.5 mg/mL lysozyme, 6 U/mL DNase I, and 1 mM PMSF) and stirred for 1 h at 4°C. Cells were disrupted by passing them three to five times through a French press (Thermo Fisher) and centrifuged at 50,000 × *g* for 1 h at 4°C. Supernatant was loaded onto a 25 mL Glutathione Sepharose 4 Fast Flow column (Cytiva) and washed with 1.5 column volumes (CV) of wash buffer I (50 mM Tris-HCl pH 7.5, 200 mM NaCl, 5% [wt/vol] Glycerol, 2 mM MgCl2, and 2 mM DTT). Subsequently, the column was washed with 1.5 CV of wash buffer II (50 mM Tris-HCl pH 7.5, 800 mM NaCl, 5% [wt/vol] glycerol, 2 mM MgCl_2_, and 2 mM DTT). The column was equilibrated with 1.5 CV of wash buffer I, and 50 μL (8 mg/mL) of the His-tagged PreScission protease was added for overnight digestion. Protein elution was undertaken with 1.5 CV of elution buffer (50 mM Tris-HCl pH 7.5, 200 mM NaCl, 5% [wt/vol] glycerol, 2 mM MgCl_2_, 2 mM DTT, and 20 mM GSH). The eluted protein (approximate mass 40 kDa) was further concentrated using a spin concentrator (10 kDa, Amicon, Millipore) and rebuffered in 20 mM HEPES pH 7.5, 200 mM NaCl, 2 mM MgCl_2_, 5% (wt/vol) glycerol, and 2 mM DTT. To quantify the concentration of purified protein in the samples, a bicinchoninic acid (BCA) assay was conducted using the BCA kit following the manufacturer’s recommendations (Sigma Aldrich).

### ATPase activity assay

The decrease in NADH absorbance, which is proportional to the rate of ATP hydrolysis, was measured continuously at a wavelength of 340 nm for 120 min at 20°C using the Synergy H1MF (BioTek) plate reader. The assay was performed in 25 mM HEPES/NaOH pH 7.5, 200 mM NaCl, 10 mM KCl, 10 mM MgCl_2_, 1 mM DTT, 5% (wt/vol) glycerol, 1.5 mM NADH, 10 U each of pyruvate kinase (PK) and lactate dehydrogenase (LDH), and 3 mM of phosphoenolpyruvate (PEP). The quantity of the sample was adjusted to achieve complete consumption of NADH within the measurement time. For WT Rep, Rep I217A, Rep I215/217A, Rep V216/I217A, and Rep K225A, we used a final concentration of 8 µM, 80 µM, 80 µM, 130 µM, and 50 µM of protein, respectively, to observe ATP hydrolysis within the measurement time. Experimental replicates were measured. The negative control contained all the reaction components except for the Rep protein. ATP hydrolysis was initiated by adding 1 mM ATP to each well.

### Determination of ATP turnover rate

A linear decrease in NADH absorbance over time was selected to calculate the ATP turnover rate for each measurement (as described above), spanning at least 15 min (79), i.e., the slope of the curve. An NADH standard curve ranging from 0.0 mM to 2.0 mM NADH was used to correlate the absorbance values at 340 nm with the respective NADH concentrations. Hence, the slope of the curve (absorbance decrease per time interval) could be transformed into NADH consumption over time (mM/min). Spontaneous oxidation of NADH observed in the negative control, i.e., a mock sample without Rep, was subtracted from the corresponding absorbance values. The slope, intercept, and R-squared values were determined by fitting a linear model using RStudio (R version 4.1.0).

### Accession numbers

DNA clones of TYLCV isolate “Alb13” *Rep* (Genebank ID: FJ956702.1) were kindly provided by Keygene (Wageningen, Netherlands). *Rep* from TYLCV isolate “Almeria” (*Begomovirus coheni* AJ489258.1) was synthesized by GenScript. Clones for the coding sequences of *AtSCE1* (At3g57870) and *AtSUMO1* (At4g26840) were previously described (71). For the Rep protein alignment, we included the Rep protein sequences from the following viruses: TGMV, tomato golden mosaic virus (*Begomovirus solanumaureimusivi* NC001507); CtLCV, cotton leaf curl virus *Rep* (*Begomovirus gossypialabadense* KC412251.1); BGMV, bean golden mosaic virus *Rep* (*Begomovirus costai* JF694454.1); CaLCV, cabbage leaf curl virus *Rep* (*Begomovirus brassicae* U65529.2); PepGMV, pepper golden mosaic virus *Rep* (*Begomovirus capsicummusivi* EF210556); SLCCNV, squash leaf curl China virus *Rep* (*Begomovirus cucurbitachinaense* KC222956.1); ACMV, African cassava mosaic virus (*Begomovirus manihotis* CAD20827.1); and BCTV, beet curly top virus (*Curtovirus betae* AAK59260.1). For the CRESS-DNA viruses that employ RCR protein alignment, we included the initiator protein NS1 and replication protein E1 sequences from CRPVK, cottontail rabbit papillomavirus (*Kappapapillomavirus 2*, P03112), and BPV1, bovine parvovirus 1 (*Bocaparvovirus ungulate 1*, P07296), respectively.

## Data Availability

All data supporting the conclusions of this study are available from the corresponding author upon reasonable request.

## SUPPLEMENTAL MATERIAL

Supplemental figures jvi.01286-25-s0001.pdf Figures S1 to S7.

Supplemental tables jvi.01286-25-s0002.docx Tables S1 to S4.

ASM does not own the copyrights to Supplemental Material that may be linked to, or accessed through, an article. The authors have granted ASM a non-exclusive, world-wide license to publish the Supplemental Material files. Please contact the corresponding author directly for reuse.
