# Supplementary material for: A SUMO interacting motif in the replication initiator protein of tomato yellow leaf curl virus is required for viral replication
Source: J Virol. 2025 Nov 10;99(12):e01286-25. doi: 10.1128/jvi.01286-25 (PMC12724345; doi:10.1128/jvi.01286-25)
Supplement: Supplemental figures — Figures S1 to S7. [file jvi.01286-25-s0001.pdf]

**Supplemental Figure 1. The SIM of Rep is conserved across CRESS-DNA viruses.**

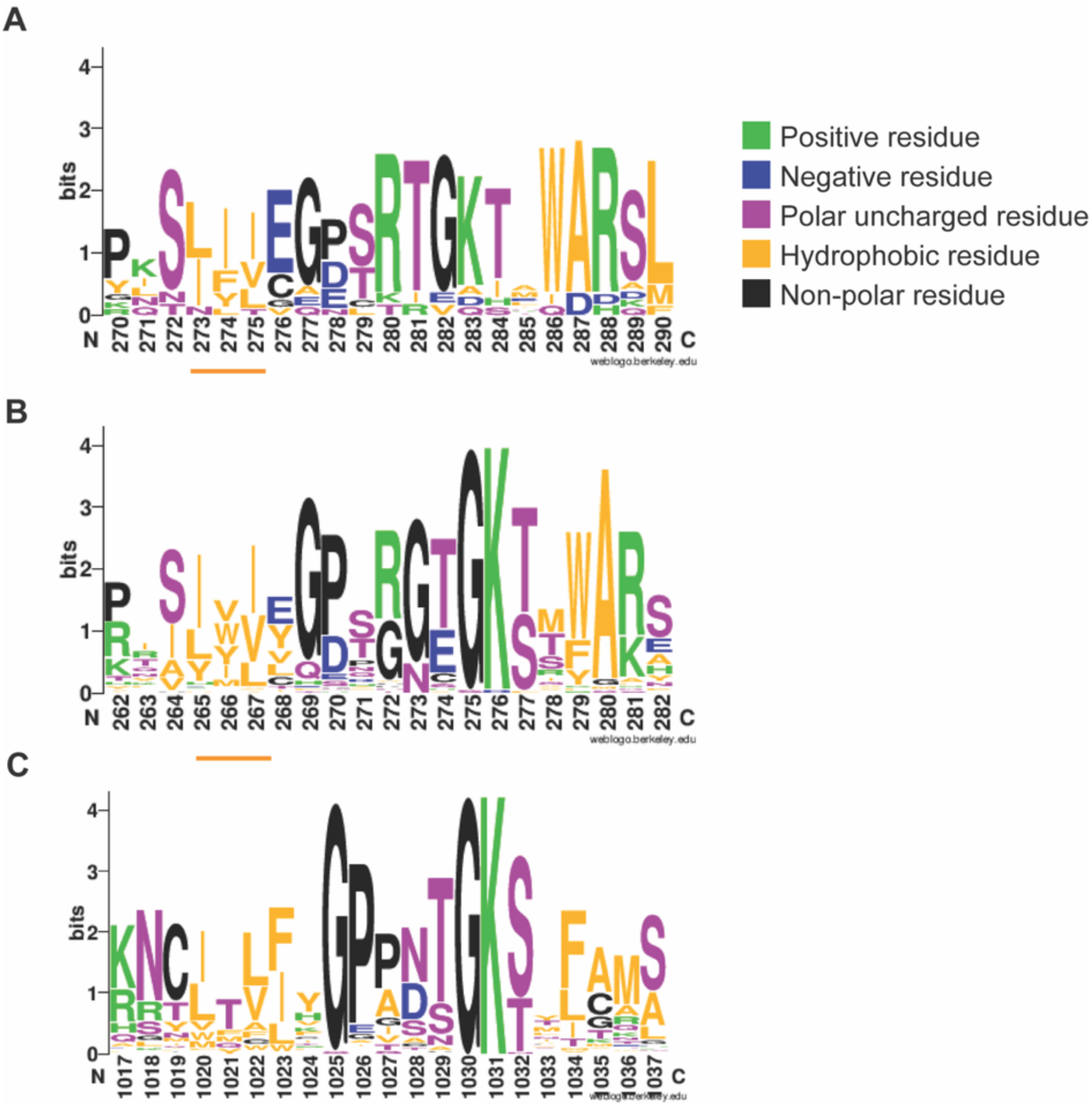


D

| Protein alignment | Panel | Conservation (%) |
|---|---|---|
| Geminiviruses | A | 60 |
| CRESS-DNA viruses | B | 50 |
| DNA viruses encoding SF3 | C | 35 |

(**A**) Consensus logo of the multiple sequence alignment (MSA) of SIM present in geminiviruses. The residues of the SIM studied are marked with an orange line (positions 273-275 in the MSA). (**B**) Similar to A, except that the logo is shown for CRESS-DNA viruses. (**C**) Similar to A, except that the logo is shown for DNA viruses that encode a SF3 helicase superfamily domain. (**D**) SIM conservation across three protein sequence alignments performed in (A-C).

**Supplemental Figure 2. The SIM mutations in Rep do not extensively alter the Alphafold structure predictions.**

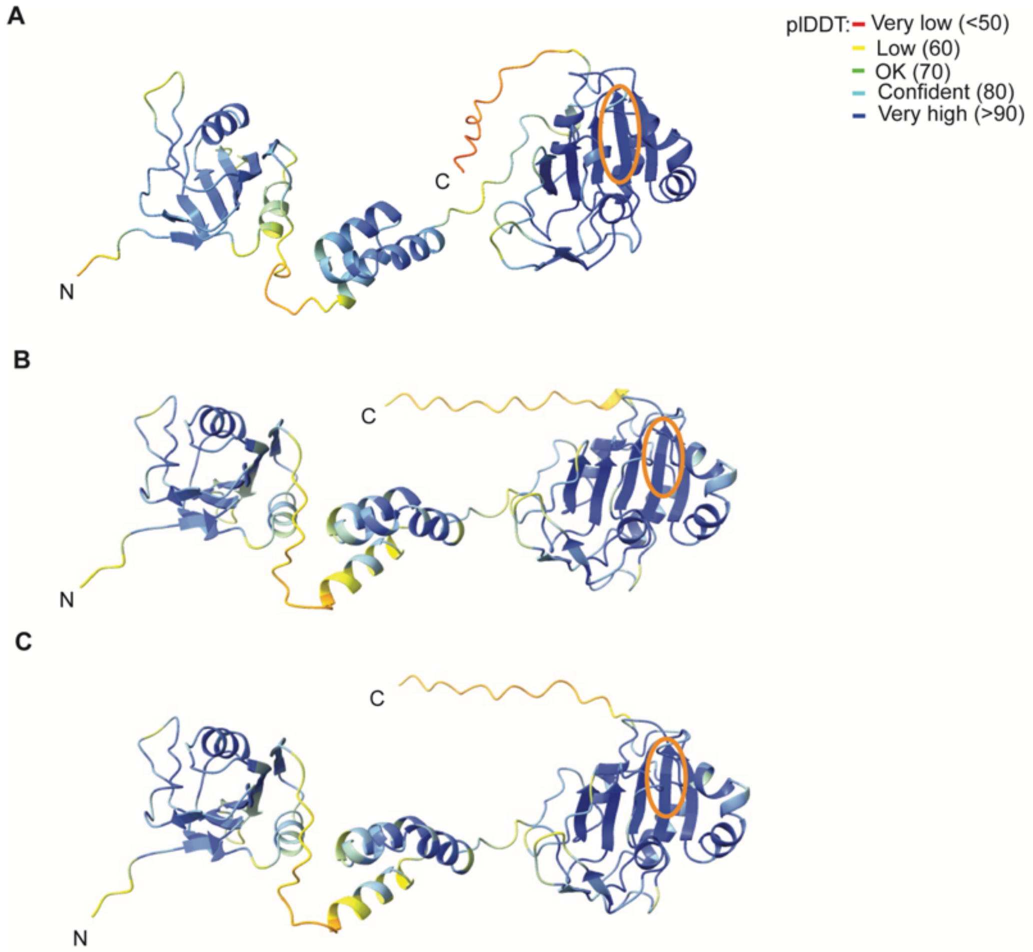


(**A**) 3D structure model of the TYLCV$_{Alm}$ Rep protein predicted by AlphaFold2. (**B**) Structure prediction of the same Rep protein using AlphaFold3 (predicted template modelling (pTM) = 0.59). (**C**) Structure prediction of Rep$^{sim}$ with AlphaFold3 (pTM = 0.59). Throughout the figure, the residues are colored by their pIDDT scores, and the SIM is highlighted by an orange ellipse; N, amino terminus; C, carboxyl terminus.

**Supplemental Figure 3. The SIM of Rep is required for the recruitment of SUMO1 and SCE1 into nuclear bodies.**

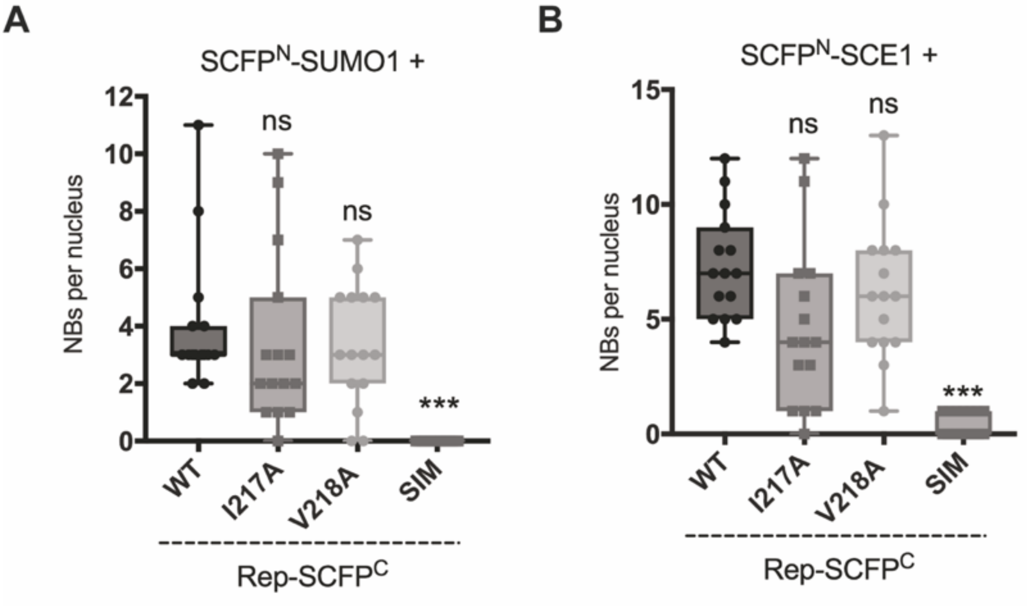


(**A**) Box plot depicting the number of NBs per nucleus in cells (n=15 cells) expressing different $Rep_{Alb13}$-SUMO1 protein pair variants, that is, WT Rep or different Rep SIM variants. In the box plot, "horizontal bars, boxes, whiskers and dots" represent "the median, interquartile ranges (IQR), data range from the minimum to the maximum, and each individual value", respectively; in the scatter plot, "the horizontal bar, whiskers and dots" indicate the "median, IQR and each individual value", respectively. A Kruskal-Wallis statistical test was performed followed by a Dunn's post-hoc test for each data set (n=15); ns: non-significant; *** $p<0.001$. Related to figure 2C. (**B**) Similar to panel A, except that different $Rep_{Alb13}$-SCE1 protein pair variants are expressed. Related to figure 2D.

**Supplemental Figure 4. Rep-SCE1 nuclear bodies are dynamic sites with SUMOylation activity.**

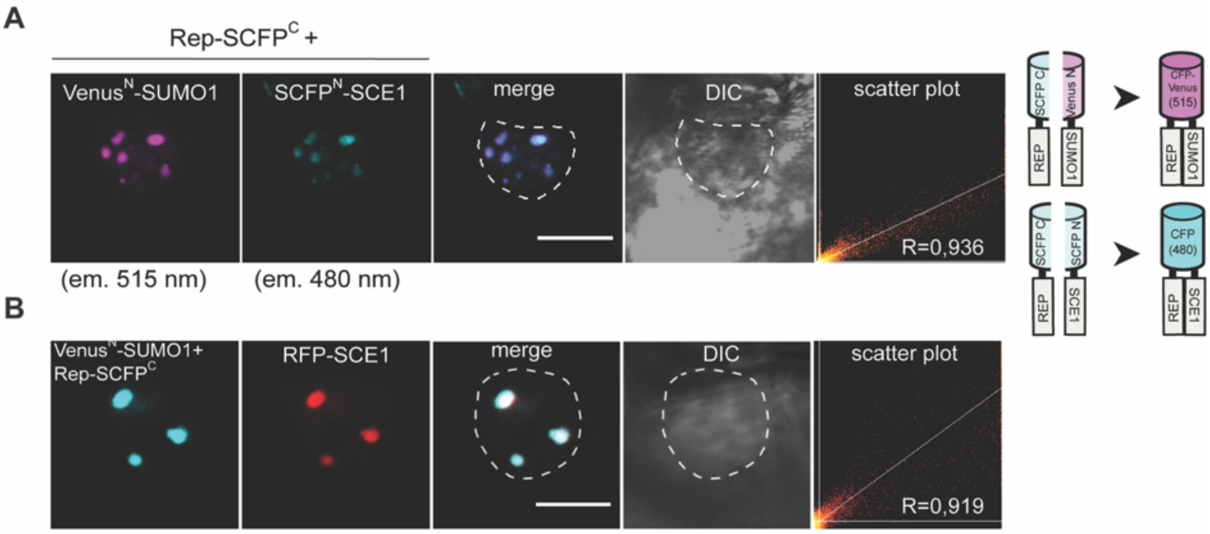


(**A**) Schematic representation of the possible interactions and the expected fluorescent emission of the multicolor BiFC (left: 515 or 480 nm) when expressing $Rep_{Alb13}$, SUMO1 and SCE1 as partners of a multicolor BiFC to allow formation of trimeric complex in NBs (right). Micrographs reveal that $Rep_{Alb13}$, SUMO1 and SCE1, when co-expressed as partners of a multicolor BiFC, co-localize and interact in a trimeric complex in NBs. (**B**) Co-localization of Rep-SUMO1 BiFC couple and RFP-SCE1 fusion in NBs in the plant nucleus. Scatter plot analysis of the pixel intensity of the CFP channel (y-axis) versus the pixel intensity of the Venus/RFP channel (x-axis) and regression correlation coefficient (using Pearson's R) of the encircled areas are shown. Scale bars represent 10 μm.

**Supplemental Figure 5. Rep from TGMV binds SUMO1 via a SIM**

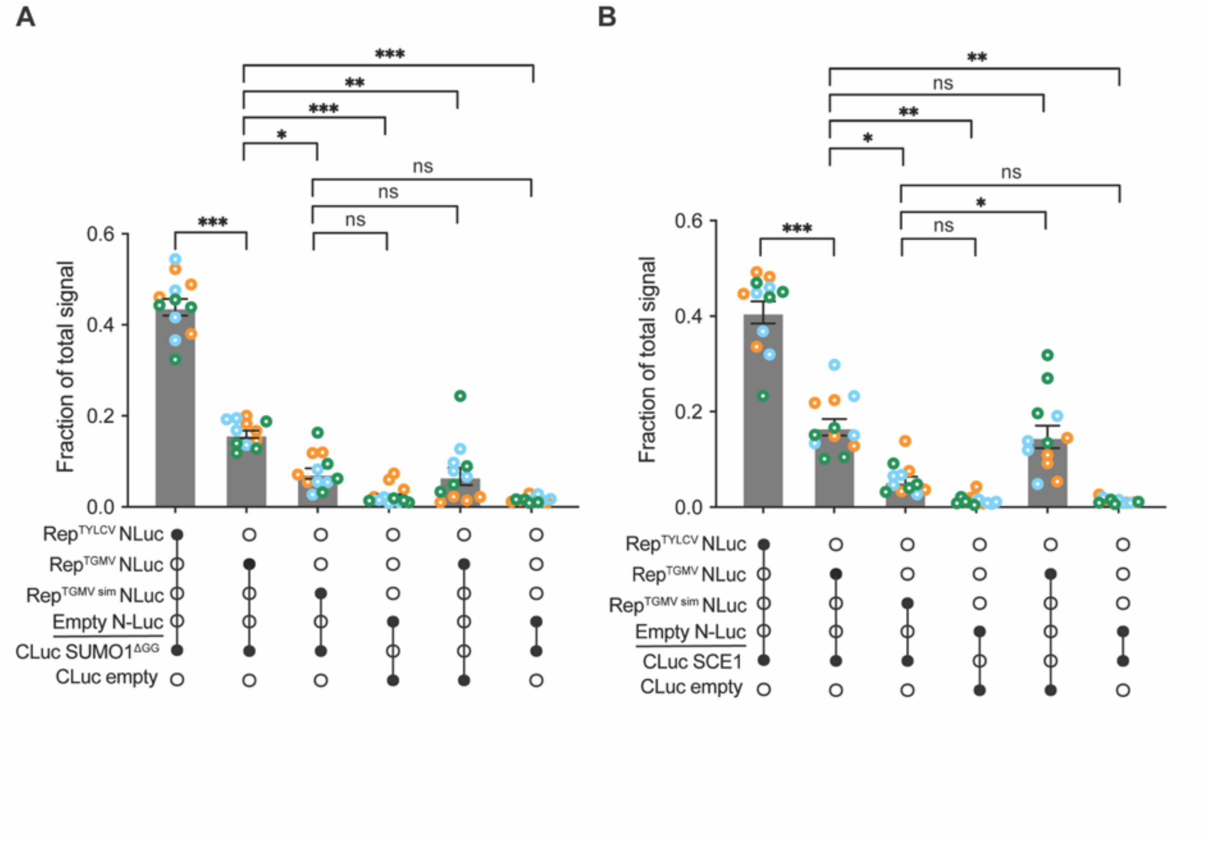


(**A**) Split-luciferase complementation assay between Rep$^{TGMV}$-SUMO1$^{\Delta GG}$ expressing either WT or Rep sim variant (I219/I220/I221A). As positive control Rep$^{TYLCV}$-SUMO1$^{\Delta GG}$ pair is included (1$^{st}$ bar). Each dot in the bar graph represents a technical replicate, while colours represent independent biological repeats. The connected black filled dots highlight the protein variants expressed for each column; Construct was fused to either the N-terminal moiety (NLuc) or C-terminal moiety (CLuc) for luciferase reconstitution. ANOVA followed by Dunnett's multiple comparison test between was performed; ns: non-significant; * p<0.05; ** p<0.01; *** p<0.001. Error bar represents SEM (n=12). (**B**) Similar to panel A, expect that Rep$^{TGMV}$-SCE1 interaction was quantified. Other details as panel A.

**Supplemental Figure 6. Rep from TYLCV SIM mutants accumulate in the nucleus.**

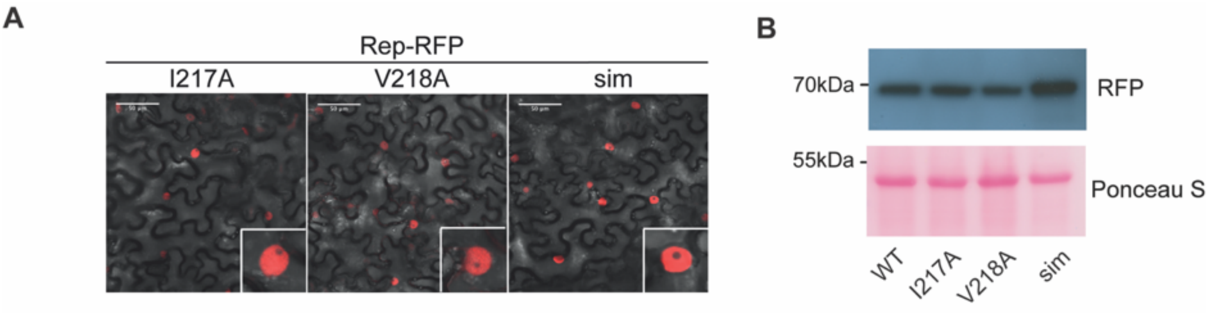


(**A**) Subcellular localization of three RFP-tagged $Rep_{Alb13}$ variants: I217A, V218A or I217/V218/I219A (sim). Images shown depict *N. benthamiana* epidermal cells upon agroinfiltration; 8× zoom of one nucleus is shown in the bottom right corner. Scale bars represent 50 μm. (**B**) Immunoblot showing the Rep-RFP protein levels (anti-RFP) in total protein extracts from agroinfiltrated *2IR-GFP N. benthamiana* leaves. To demonstrate equal protein loading, Ponceau S staining of the membranes is shown.

**Supplemental Figure 7. The SIM of Rep is required for TYLCV infectious clone replication.**

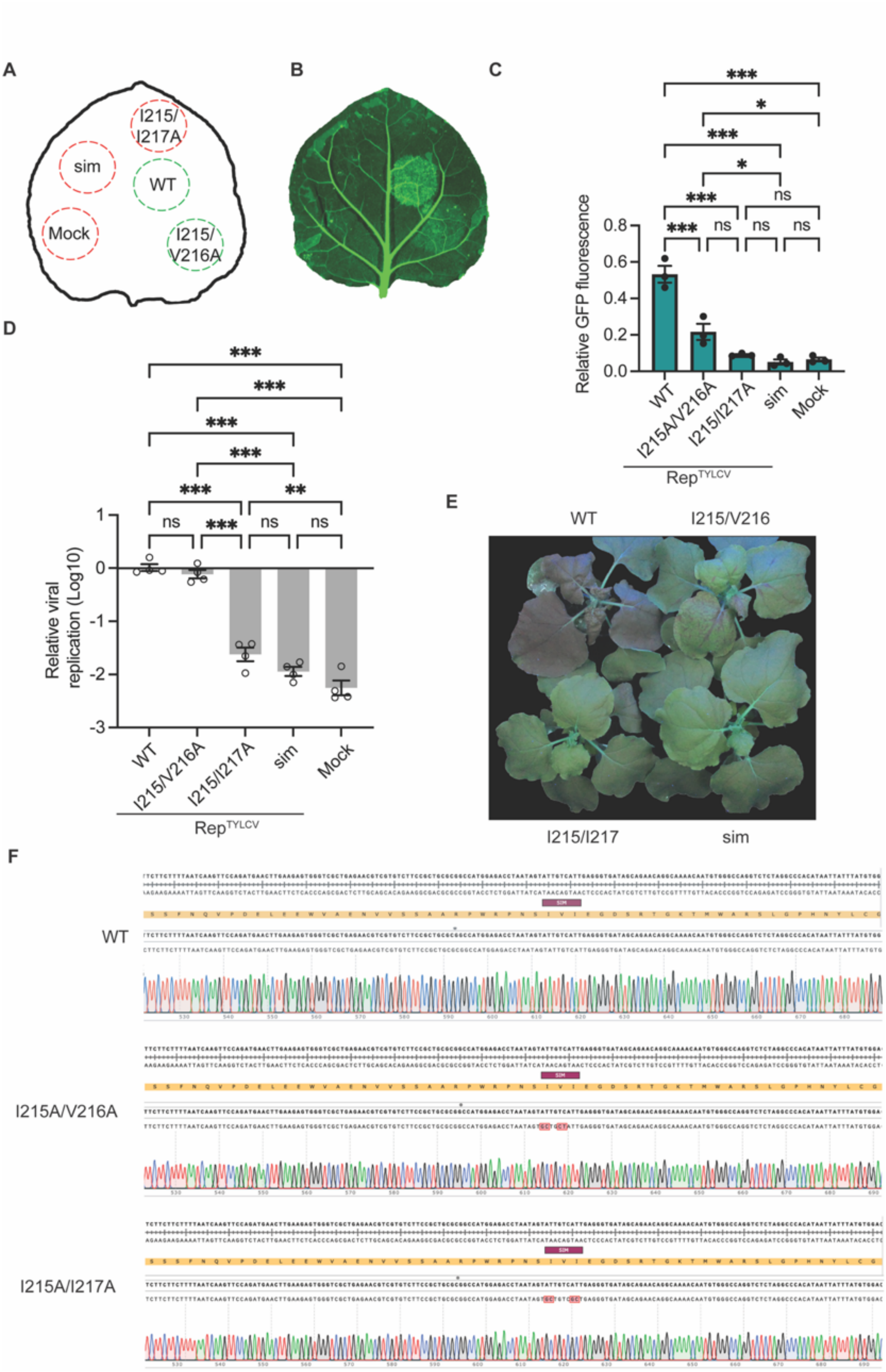

(**A**) Scheme depicting agoinfiltration of TYLCV infectious clones, WT and three different Rep SIM variants alongside the empty vector control (Mock). (**B**) Fluorescence image depicting GFP accumulation as a proxy for TYLCV replication in the reporter line *2IR-GFP N. benthamiana*. (**C**) GFP quantification of fluorescence signal in infiltrated leaves*.* ANOVA followed by Dunnett's multiple comparison test between was performed; ns: non-significant; * $p<0.05$; ** $p<0.01$; *** $p<0.001$. Error bar represents SEM (n=3). (**D**) Quantification of viral titres using real-time PCR on the extracted DNA from the apical leaves. Levels were normalized to the viral titres by the wildtype virus ($Log_{10}=0$). Other details as panel C. Error bar represents SEM (n=4). (**E**) GFP fluorescence pictures of the *2IR-GFP N. benthamiana* depicted in **Fig. 4A**. (**F**) Representative Sanger sequencing read on TYLCV amplicon from an expanded TYLCV-infected near the shoot apex (**Fig. 4B**).
