# Supplementary material for: A SUMO interacting motif in the replication initiator protein of tomato yellow leaf curl virus is required for viral replication
Source: J Virol. 2025 Nov 10;99(12):e01286-25. doi: 10.1128/jvi.01286-25 (PMC12724345; doi:10.1128/jvi.01286-25)
Supplement: Supplemental tables — Tables S1 to S4. [file jvi.01286-25-s0002.docx]

**Supplemental Table 1. Position of the hydrophobic residues in the Reps studied**

| Rep variant  (NCBI ID) | Rep^Alm^ (AJ489258.1) | Rep^Alb13^ (FJ956702.1) | Rep^TGMV^ (NC001507) |
| --- | --- | --- | --- |
| **Position 1** | I215 | I217 | I219 |
| **Position 2** | V216 | V218 | I220 |
| **Position 3** | I217 | I219 | I221 |

**Supplemental Table 2. Sequence of the primers used in this study**

| Name | Sequence 5’ to 3’ |
| --- | --- |
| Rep FW_attB1 | GGGGACAAGTTTGTACAAAAAAGCAGGCTTGAGAGACTCCGATTGACCAAG |
| Rep Rv_attB2 | GGGGACCACTTTGTACAAGAAAGCTGGGTAGTCTTCGCTATGCGGTGTTG |
| Rep Rv_attB2 STOP | GGGGACCACTTTGTACAAGAAAGCTGGGTACTAGTCTTCGCTATGCGGTGT |
| REPattB1 Rv short NO stop | AGAAAGCTGGGTCGTCTTCGCTATGCGGTGTT |
| TYCRepFwGW | A AAA GCA GGC T CC ATGCCTCGTTTATTTAAAATATATGC |
| TYCRepRvGWns | A GAA AGC TGG GT C CGCCTTATTGGTTTCTTC |
| TY FM REP sim RV | TTCTGCTATCACCCTCAGCGGCAGCACTATTAGGTCTCCATGGCCGCGCAGC |
| TY FM REP sim FW | GCTGCGCGGCCATGGAGACCTAATAGTGCTGCCGCTGAGGGTGATAGCAGAA |
| TY Rep I215A | GAGACCTAATAGTgcTGTCATTGAGGGTG |
| TY Rep I215A | CACCCTCAATGACAgcACTATTAGGTCTC |
| TY Rep V216A | GACCTAATAGTATTGcCATTGAGGGTGATAG |
| TY Rep V216A | CTATCACCCTCAATGgCAATACTATTAGGTC |
| TY Rep I217A Fw | CTAATAGTATTGTCGCTGAGGGTGATAGCAGAA |
| TY Rep I217A Rv | GCTATCACCCTCAGCGACAATACTATTAGGT |
| TY Rep V216A/I217A Fw | GACCTAATAGTATTGCCGCTGAGGGTGATAGCA |
| TY Rep V216A/I217A Rv | GCTATCACCCTCAGCGGCAATACTATTAGGTC |
| Nb25SrRNAFw | ATAACCGCATCAGGTCTCCA |
| Nb25SrRNARv | CCGAAGTTACGGATCCATTT |
| MECs2Fw | CTGGGGACCTGCAGGC |
| MECs2Rv | CGCGCGGTGTCATCTATGTTAC |
| FattB1_SCE1 | GGGGACAAGTTTGTACAAAAAAGCAGGCTCAATGGCTAGTGGAATCGCTCGTGG |
| R_attB2_SCE1(stop) | GGGGACCACTTTGTACAAGAAAGCTGGGTCTTAGACAAGAGCAGGATACTG |
| LBb1.3 | ATTTTGCCGATTTCGGAACCACCATC |
| Ubq10_End_Fw3 | TTAGTTTCTAGTTTGTGCGATCG |
| Gateway Site Reverse | AACCACTTTGTACAAGAAAGCTG |
| RepTyCzK225AFw | GGTGATAGCAGAACAGGCgcAACAATGTGGGCCAGGTC |
| RepTyCzK225ARv | GACCTGGCCCACATTGTTgcGCCTGTTCTGCTATCACC |
| RTCPTYLCD-F | GAAGGCTGAACTTCGACAGC |
| RTCPTYLCD-R | GGACTTTACATGGGCCTTCAC |

**Supplemental Table 3. Plasmids used in this study**

| Gene name | Backbone | Template/source | Primers | Procedure |
| --- | --- | --- | --- | --- |
| TYLCV Alb13 Rep | pENTR207 | KeyGene N.V. |  |  |
| TYLCV-Almeria Rep | pENTR207 | E.R. Bejarano |  |  |
| SUMO1 | pENTR221 | (71) |  |  |
| SCE1 | pENTR-TOPO | (71) |  |  |
| SCE1 ^SUM1^ | pENTR221 | (63) |  |  |
| TYLCV Rep Alb13 SIM | pENTR207 | TYLCV Alb13 Rep | 7694/7695 | Quikchange |
| TYLCV-Almeria Rep SIM | pENTR207 | TYLCV-Almeria | 7694/7695 | Quikchange |
| TYLCV-Almeria Rep I215A | pENTR207 | TYLCV-Almeria | 8500/8501 | Quikchange |
| TYLCV-Almeria Rep V216A | pENTR207 | TYLCV-Almeria | 8502/8503 | Quikchange |
| TYLCV-Almeria Rep I217A | pENTR207 | TYLCV-Almeria | 10186/10187 | Quikchange |
| TYLCV-Almeria Rep I215A/V216A | pENTR207 | TYLCV-Almeria | 7368/7370 | Cloning from infectious clone |
| TYLCV-Almeria Rep I215A/I217A | pENTR207 | TYLCV-Almeria | 8618/8619 | Quikchange |
| TYLCV-Almeria Rep V216A/I217A | pENTR207 | TYLCV-Almeria | 7368/7370 | Cloning from infectious clone |
| TYLCV-Almeria Rep I215/V216A/I217A | pENTR207 | TYLCV-Almeria | 7368/7370 | Cloning from infectious clone |
| TYLCV-Almeria Rep K225A | pENTR207 | TYLCV-Almeria |  | Quikchange |
| pDEST-gw-SCYCE | pDEST-^GW^SCYCE | (Gehl et al. 2009) |  |  |
| pDEST-SCYNE(R)-gw | pDEST-SCYNE^GW^ | (Gehl et al. 2009) |  |  |
| pGBKT7-DEST | pGBKT7 | (Chien et al. 1991) |  |  |
| pGADT7-DEST | pGADT7 | (Chien et al. 1991) |  |  |
| pMet-gw-cUb-URA1 | pMet | T. Liebrand |  |  |
| pCup-nUb-gw-CYC1 | pCup | T. Liebrand |  |  |
| 35S-gw-mRFP | pGWB654 | (Nakamura et al. 2010) |  |  |
| TYAlm WT | PUC57 | GenScript |  |  |
| TYAlm I215/V216A/I217A | PUC57 | GenScript |  |  |
| TYAlm I215A/V216A | PUC57 | GenScript |  |  |
| TYAlm I215A/I217A | PUC57 | GenScript |  |  |
| P19 inhibitor silencing | pBIN61 |  |  |  |
| HA-RedFFLuc Cterm-Gateway box | pGWB402 |  |  |  |
| Gateway box -RedFFLuc Nterm-FLAG-Stop | pGWB402 |  |  |  |
| Gateway box -RedFFLuc Cterm-HA-Stop | pGWB402 |  |  |  |
| Rep TY Alb13 - RedFF Nterm FLAG GWB402 | bgIFP5594 |  |  |  |
| HA RedFF Cterm SUMO1deltaGG pGWB402 | bgIFP5595 |  |  |  |

**Supplemental Table 4. Antibodies used in this work.**

| Antibody | Mono/polyclonal | Organism | Origin | Dilution |
| --- | --- | --- | --- | --- |
| αGFP | Monoclonal | Rat | Chromotek [3H9] | 1:12000 |
| αRFP | Monoclolal | Mouse | Chromotek [6G6] | 1:10000 |
| αRat-HRP | Polyclonal | Goat | Pierce (31470) | 1:10000 |
| αMouse-HRP | Polyclonal | Goat | Pierce (31430) | 1:10000 |
